# *CgAS*, a gene encoding anthranilic acid synthase, contributes to tryptophan biosynthesis and enhanced chaetoglobosin A production in *Chaetomium globosum* W7

**DOI:** 10.1016/j.synbio.2025.05.001

**Published:** 2025-05-06

**Authors:** Shanshan Zhao, Zefei Wang, Liyan Tian, Kejing Li, Shiwei Sun, Gen Chen, Daoqiong Zheng

**Affiliations:** a Donghai Laboratory, Zhoushan, Zhejiang 316021, China; b Institute of Marine Biology and Pharmacology, Ocean College, Zhejiang University, Zhoushan, 316021, China; c School of Life Science and Technology, Harbin Institute of Technology, Harbin, 150080, Heilongjiang province, China

**Keywords:** Chaetoglobosin A, *Chaetomium globosum* W7, Anthranilic acid synthase, Tryptophan, Metabolome

## Abstract

Chaetoglobosin A (cheA) is a complex indole alkaloid exhibiting preferential cytotoxicity against plant pathogens, parasites, and tumor cells. However, the limited production and high synthesis costs of cheA impede its widespread application. Tryptophan serves as a precursor for cheA biosynthesis, and strategic modification of the expression of key genes represents a novel approach to enhance the target yield. Herein, *CgAS*, a gene encoding anthranilic acid synthase involved in tryptophan synthesis, was identified through bioinformatics analysis and overexpressed via a promoter optimization strategy in *Chaetomium globosum* W7. The AS1 and AS3 mutants, in which the *CgAS* gene was constitutively overexpressed under the control of promoter *oliC*, presented a significant increase in tryptophan accumulation. *CgAS* overexpression caused a dramatic increase in cheA production, reaching a maximum yield of 217.81 mg/L during the stationary phase, which was 3.73-fold higher than that noted in the wild-type strain. Interestingly, AS1 and AS3 mutants exhibited a substantial upregulation in the transcription levels of critical genes involved in cheA biosynthesis. Phenotypic characterization and metabolomic analysis indicated that tryptophan accumulation strengthened microbial nitrogen metabolism, which not only provided sufficient precursors for secondary metabolism, but also functioned as an essential energy source to accelerate fungal development and sporulation. These findings illustrate the impact of precursor accumulation on indole alkaloid biosynthesis and provide novel insights for optimizing the production of biopesticides and clinical drugs.

## 1 Introduction

Members of the genus *Chaetomium* are ubiquitously distributed around the world in various ecosystems [1,2], as one of the largest genera of saprophytic ascomycetes, *Chaetomium* is considered as the microbial cell factory for the production of structurally unique and complex bioactive secondary metabolites possessing various biological activities, such as antineoplastic, antimicrobial, cytotoxic, and antioxidant activities [[3], [4], [5]]. Among them, cheA, initially identified by Seklta et al. [6], is the most abundant cytochalasan predominantly biosynthesized by *C. globosum*. As a typical compound of the cytochalasan family, cheA exhibits preferential cytotoxic activity against chronic lymphocytic leukemia (CLL) cells. CLL is an incurable disease with a high recurrence rate, and cheA can induce cell-cycle arrest and inhibit membrane ruffling and cell migration by targeting filamentous actin in the CLL cells [7]. Furthermore, cheA has been found to show persistent cytotoxic activities against colorectal cancer (CRC) cells, with IC_50_ values below 5 μΜ after 24-h of treatment. In CRC cells, cheA targets the Keap1-Nrf2-HO-1 pathway to induce reactive oxygen species (ROS) generation and JNK1/2 phosphorylation, eventually triggering apoptosis [8]. In addition, cheA has also presented remarkable efficacy in the prevention and treatment of multiple pathogens, including *Xanthomonas oryzae*, *Setosphaeria turcica* and *Fusarium sporotrichioides* [9,10]. Thus, cheA is a promising therapeutic and biological control agent worthy of further research to boost biomedical and sustainable agricultural development.

The genetic and molecular framework for cheA biosynthesis has been proposed and identified in *Penicillium expansum* by using RNA-mediated gene silencing strategies [11]. In the initial stage of cheA biosynthesis, the monomeric compound consisting of acetate and malonate is polymerized under the catalysis of a PKS-NRPS hybrid megasynthetase to generate a nonaketide chain. During this process, a standalone enoyle reductase (ER), marked within the cluster, functions as a chaperone protein and plays a pivotal role in carbon-chain elongation [12]. Subsequently, the carbon backbone is condensed with an activated tryptophan to yield the required precursor isoindolone-fused macrocycle (prochaetoglobosin I) through the intramolecular Diels-Alder cyclization [13]. In the final stage of cheA biosynthesis, three distinct oxygenase catalyze the conversion of the non-functional structure into the terminal product, cheA [12]. Given the substantial research and commercial significance of cheA, many exhaustive studies on cheA biosynthesis have been conducted to improve its production. For instance, by using RNAi-mediated silencing strategy, Hu et al. identified a heterotrimeric Gα-cAMP/PKA that positively modulated cheA production in *C. globosum* [14]. Furthermore, several transcriptional regulators, including CgcheR and CgLaeA, have been demonstrated to influence cheA biosynthesis either in a pathway-specific or non-targeted manner. The modulation of these gene expressions through molecular biology techniques facilitates the biosynthesis of the target products [15,16]. Despite these efforts to improve cheA production, the yields of cheA remain considerably lower than those of certain well-exploited biological drugs. Therefore, it is imperative to identify the factors that restrict cheA biosynthesis and develop strategies to improve the cheA-producing capacity of *C. globosum* W7.

Acetyl-CoA, malonyl-CoA, and multi-amino acids (such as tryptophan, glutamate, glutamine, and serine), which are mainly derived from numerous essential primary metabolic pathways, serve as the starting element, extender, and crucial building modules for polyketide biosynthesis, respectively. Owing to the decline in primary metabolism at the stationary phase of fermentation, polyketide production is generally limited by the availability of these essential precursors [17,18]. Hence, redesigning or optimizing the pathways for the synthesis of precursors is critical for the enhancement of secondary metabolite yields. A previous study found that overexpression of acetyl-CoA carboxylase, achieved by replacing the native promoter with a strong constitutive initiator *PadhA*, in *Aspergillus terreus* (ATCC 20542) resulted in a marked improvement in lovastatin production, which was 140 % higher than that noted in the wild-type strain [19]. A genetic analysis suggested that the *pdh* and *dahp* genes, located at the periphery of the balhimycin biosynthetic cluster, encode the primary metabolic enzymes responsible for the precursor supply of tyrosine, and that additional copies of the *dahp* gene significantly increased specific glycopeptide productivities by approximately threefold [20]. Furthermore, it has been found that the *dahp*-paralogous genes are conserved and occur in the phosphinothricin biosynthetic gene cluster of *Streptomyces* spp. [21]. Thus, manipulation of precursor supply could be a promising approach to promote the yield of target natural metabolites, and might be effective in increasing antibiotic production levels.

The objective of the present study was to improve the cheA yield by increasing the precursor supply. As a synthetic substrate for the target product, tryptophan has a crucial role in cheA biosynthesis. Accordingly, the *CgAS* gene of *C. globosum* W7, which encodes anthranilic acid synthase (AS) involved in the tryptophan biosynthesis pathway, was identified by bioinformatics analysis and overexpressed though promoter optimization strategy. Subsequently, the impact of *CgAS* on tryptophan and cheA production was monitored by HPLC, with the wild-type strain utilized as the control. Given that tryptophan also functions as an important nutrient and energy source, the phenotypic properties, fungal development, and metabolic differences between *C. globosum* W7 and other *CgAS*-modified strains were further compared. The findings of the present study provide a potential strategy to optimize antibiotic production.

## 2 Materials and methods

### 2.1 Strains, plasmids, culture media, and growth conditions

*Chaetomium globosum* W7 (preservation number CGMCC 3.14974), a promising biocontrol filamentous fungi capable of producing chaetoglobosin A, was used as the wild-type strain in this study. *Escherichia coli* DH5α and *Trans*DB3.1 competent cells were employed for genetic transformation and plasmid propagation. Other species involved in this study are summarized in Table S1. The *E. coli* strains and its derivatives were incubated in Luria-Bertani or low salinity Luria-Bertani medium (LB culture comprised of 10 g tryptone, 5 g NaCl and 5 g yeast extraction per litre, with a pH of 7.2), because of the presence of salinity-sensitive genes on the carrier. Where appropriate, experiments in petri dishes were made with the same media supplemented with suitable concentrations of antibiotics for cloning purposes. For fungal growth and morphological comparison, *C. globosum* W7 (the wild-type species) and its mutants were cultivated at 28 °C statically or shaken at 180 rpm on PDA (potato 200 g, dextrose 20 g, and 1000 mL water) medium supplemented with hygromycin B (200 μg/mL) and agar (15 g/L) when required. Besides, for cheA and tryptophan yield detection, the prepared spore suspensions of strains *C. globosum* W7 and its derivatives were transferred into 50 mL PDA broth in 150 mL flasks at 28 °C, and samples were taken and measured at various time points of equal intervals. For transformation of protoplasts, protoplast regeneration medium, in which mixed with protoplasts-genetic modification vector and mediator (PEG4000), was composed of yeast extract and peptone 0.1 g, sucrose 3.42 g, agar 0.15 g and 10 mL water. PDA semisolid medium (1 % agar) covered with hygromycin (200 μg/mL) was used as screening plate of gene-modified mutants.

### 2.2 In silico analysis of CgAS and construction of the phylogenetic tree

According to the BLASTP algorithm performed on the NCBI database (National Center for Biotechnology Information), CgAS (GenBank: XP_001221698.1), which was identified as the gene encoding the anthranilic acid synthase in *C. globosum* W7, has a 2376-base pair (bp) open reading frame that encodes a polypeptide of 770 amino acids. Orthologous proteins and similarity analysis were conducted using the NCBI database. The secondary structure elements were predicted by SWISS-MODEL [22,23], and multiple alignment was aligned by using the biotool Web servers ESPript 3.0 (https://espript.ibcp.fr/ESPript/cgi-bin/ESPript.cgi) [24]. Additionally, a phylogenetic tree was constructed by the neighbor-joining (NJ) distance algorithm with the Jones-Taylor-Thornton (JTT) model [25] using employing Molecular Evolutionary Genetics Analysis (MEGA) software version 7.0 [26,27]. The bootstrap resampling analysis with 1000 replicates was performed to evaluate the stability of clades and the tree topology [28], and the Chiplot online tool (https://www.chiplot.online/) was employed for the final visualization and ornament [29]. The conserved region of the CgAS was analyzed by the Simple Modular Architecture Research Tool (SMART) (http://smart.embl-heide lberg.de/) and Conserved Domains (https://www.ncbi.nlm.nih.gov/cdd/).

### 2.3 Plasmid constructions

All plasmids and oligonucleotides utilized in this study are presented in Supplementary Tables 1 and 2, respectively. To verify promoter strength in *C. globosum* W7, green fluorescence and hygromycin resistance markers were used as reporter genes. The promoter sequence were listed in Table S3. Taking pSK-*gpdA*-HygREG as an instance, the construction procedure was as follows: the *gpdA* constitutive promoter, which was derived from *A. oryzae*, was amplified using the primer pair gpdA-F/gpdA-R, the primers gpHygRF/gpHygRR and gpdA-EGF/gpdA-EGR were designed to obtain the sequence of the *HygR* and *EGFP* genes, respectively. Subsequently, the linearized backbone of pBlueScript-SK, digested with *Hin*dIII and *Eco*RI*,* was ligated with the target fragment containing 20–25 bp homologous regions on both ends, following the protocol outlined in the ClonExpress MultiS One Step Cloning Kit (Vazyme). The resultant plasmid was then transformed into *E. coli* DH5α competent cells to produce the verification plasmid pSK-*gpdA*-HygREG. For the purpose of regulator overexpression, *CgAS* was amplified via PCR using the oligonucleotides primers oliC-AS-F2/oliC-AS-R2. The backbone carrier was pCR-Blunt-*HygR*, in which a kanamycin resistance gene was replaced by *HygR* in pCR-Blunt. For the construction of pHygR-*oliC*-*AS*, the linearization plasmid was generated by digesting with *Bam*HI and *Hin*dIII, then connected with the optimized promoter and *CgAS* though recognizing the homologous region using the ClonExpress MultiS One Step Cloning Kit (Vazyme).

### 2.4 Transformation of C. globosum W7 protoplasts

Following the construction the plasmids pSK-*gpdA*-HygREG, pSK-*trpC*-HygREG, pSK-*oliC*-HygREG and pHygR-*oliC*-*AS*, PEG4000 mediated plastid transformation was employed to transform *C. globosum* W7, facilitating gene modification and expression level detection. The operational procedures was performed as previously described [30,31]. Transformational strains were selected based on their resistance to hygromycin (200 μg/mL). Following screening and purification, all genetically modified products were maintained on PAD medium for spore collection and preserved as glycerol suspensions (50 %, v/v) at −80 °C.

### 2.5 Morphological observation and nuclear staining analysis

*C. globosum* wild type and its derivatives were cultured at a constant temperature, with growth and conidiation monitored over time. For the enumeration of *C. globosum* spores, all tested species were inoculated in Oxford cups (each inoculated with the same concentration spores) on PDA medium for 2, 4 and 6 days. The colony diameter and sporulation quantity were measured by vernier caliper and blood counting chamber, respectively. Morphology properties of the *C. globosum* W7, AS1 and AS3 were observed by light microscopy (Nikon ECLIPSE E200) and scanning electron microscopy (SEM). Specimens for SEM were prepared following the protocol described by Wang et al. [32], and details was described as follows. A square (0.5 cm in length) was excised from the agar plate of tested species, and then fixed in a 2.5 % glutaraldehyde buffer (pH 7.2) at 4 °C for approximately 1.5 h. Subsequently, after washing twice with phosphate buffer, samples were further treated with a graded series of ethanol for dehydrating. Dried using a lyophilizer, coated with a gold layer, and examined in TESCAN VEGA 3 SEM. To identify the number of the cell nuclei in cell septa, the mycelia and spores were stained with 10 μg/mL 4,6-diamidino-2-phenylindole (DAPI, Sigma-Aldrich, USA) for 10 min, and observed utilizing a fluorescence microscope (Leica DM6 B). Experiments were performed in triplicate.

### 2.6 Determination of mycelial biomass and detection the yield of chaetoglobosin A and tryptophan

Approximately 1 × 10^7^ spores of the wild-type species, AS1 and AS3, in which a essential gene involved in tryptophan biosynthesis was overexpressed by employing a promoter optimization strategy, were inoculated into Fernbach flasks, with each flask containing fifty-millilite PDA liquid medium. Fermentation was carried out at 28 °C and sampled at three days intervals. To quantify the mycelial biomass, 50-mL cell cultures that cultivated in PDA liquid medium were initially subjected to centrifugation at 12,000 rpm for 5 min to separate the medium. The resulting pellet was resuspended in sterile deionized water and centrifuged again under the same conditions to eliminate residual medium. This procedure was repeated three times. The biomass was then transferred to a Büchner funnel equipped with sterile filter paper, collected via vacuum filtration, and dried at 60 °C to a constant weight to determine the dry cell weight. For determination of chaetoglobosin A (cheA) production, samples were first centrifuged at 12,000 rpm for 10 min to remove the cells, and then extracted with equal volumes of ethyl acetate (EtOAc) which was shacking treatment overnight. Dehydrated with anhydrous sodium sulfate and then enriched in a rotary evaporator at 35 °C. Subsequently, the extractives of all determining samples were then subjected to reversed-phase high-performance liquid chromatography (HPLC) analysis using a Waters 2695-2489 system equipped with a TC-C18 column (Agilent, 4.6 mm × 250 mm, 5 μm) on an isocratic elution condition of 45 % CH_3_CN (v/v) in H_2_O at a flow rate of 1.0 mL/min, and the detection wavelength was 227 nm [16]. CheA (C_32_H_36_N_2_O_5_) standard used as the control was purchased from Sigma-Aldrich (Germany). For the tryptophan concentration assay, the fermentation mixture underwent centrifugation at 12,000 rpm for 10 min at 4 °C to remove fungal cells and impurities. The supernatant was then filtered though a 0.22 μm polyethylene filter, and measured by the Waters 2695-2489 system with TC-C18 column on an isocratic elution condition of 10 % CH_3_CN (v/v) in KH_2_PO_4_ (8 mmol/L) at a flow rate of 1.0 mL/min at 35 °C, and the detection wavelength was 280 nm. These experiments were all performed in triplicate.

### 2.7 RNA isolation and quantitative real-time PCR

*C. globosum* W7 and its mutants were cultured in PDA liquid medium at 28 °C, with samples collected at regular intervals. The harvested mycelium samples were frozen in liquid nitrogen, and ground into a powder for RNA extraction using the RNA prep pure plant kit (Tiangen, China). RNA quality was analyzed via agarose gel electrophoresis, and the concentration was measured with a NanoDrop instrument. Single-stranded cDNA was synthesized with the EasyScript First-Strand cDNA Synthesis SuperMix (TransGen Biotech, China). The expression of candidate genes was analyzed using Real-time PCR System with the Trans Start Top Green qPCR SuperMix (TransGen Biotech, China). The relative abundances of critical genes associated with cheA biosynthesis were normalized to the expression levels of the endogenous reference gene *β*-actin (GenBank Accession No. CH408033.1). Specific paired primers involved in this research was listed in Table S2. Using the 2^−ΔΔCT^ method, the relative quantification of each target transcript were calculated automatically [33]. All assays were conducted in triplicates and each experiment was also repeated three times.

### 2.8 Metabolome analysis

The wild-type species *C. globosum* W7 and its derivatives were singly cultured in 50 mL of sterile PDA broth and fermented at 180 rpm and 28 °C for 5 days, respectively. Mycelial cells were collected by centrifugation at 6000 rpm for 10 min, discarded the supernatant. For purpose of removing the medium attached to the cells surface, the sediments were washed with PBS salt ionized buffer until the extracellular substances solvent becomes clear or did not appear colored material. Cells were rapidly quenched with liquid nitrogen and subjected to an acidic environment to minimize oxidation. Intracellular metabolites were extracted from fungal cells employing a method previously described by Tu et al. [34]. Cellular metabolites were quantitated by LC/MS system, which is composed of Acquity UPLC I-Class PLUS tandem Xevo G2-XS QTof of high resolution mass spectrometer (Waters Corporation), with a UPLC HSS T3 column (Waters, 2.1 mm × 100 mm, 1.8 μm). The eluents for the positive polarity mode and negative polarity mode were eluent A (0.1 % formic acid aqueous solution) and eluent B (0.1 % formic acid acetonitrile). The pathways of metabolites were analyzed with the databases of KEGG (http://www.kegg.jp) and MetaboAnalyst (http://www.metaboanalyst.ca/) [35]. T test was used to calculate the difference significance pvalue of each compound in various detective groups. The difference metabolites of KEGG pathway enrichment significance were calculated using hypergeometric distribution test.

### 2.9 Statistical analysis

All data are shown as the mean ± SEM and were analyzed using unpaired *t*-test. For statistical studies, one-way ANOVA with Tukey's post hoc comparisons test were performed. Three biological replicates for each treatment were conducted for the statistical analysis in this article. The asterisks in the figures denoted their significant differences as follows: ∗p < 0.05, ∗∗p < 0.01. Primer 10.0 (GraphPad Software, San Diego, CA) and Origin (OriginLab, Northampton, Massachusetts, USA) were utilized for all statistical analyzed and graphs generated.

## 3 Result

### 3.1 Characteristics of the CgAS gene in C. globosum W7

**Fig. 1:**
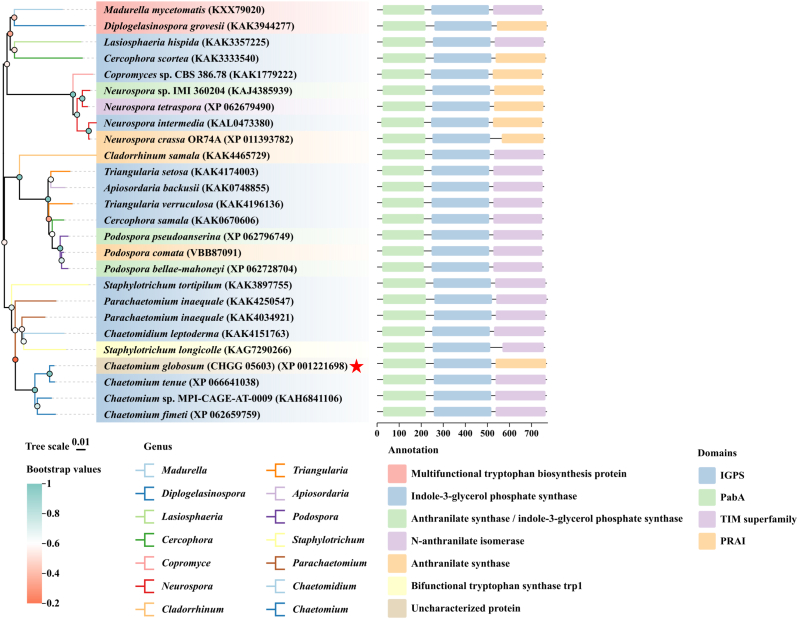
Phylogenetic tree and domain architecture of CgAS and its similarity organisms based on the amino acid analysis. The ClustalW algorithm was used to perform a sequence alignment, which was used to reconstruct the tree using the Neighbor-joining method and a JTT matrix-based model. The bootstrap value of trees in which the associated taxa clustered together is shown in circles with various colors.

BLASTP algorithm analysis conducted on the online platform NCBI identified *CgAS* sequence (XP_001221698.1), a putative AS-encoding gene, from the *C. globosum* W7 genome database. Preliminary studies of the sequence revealed that the open reading frame (ORF) of the *CgAS* gene is 2376 nt in length, comprising one intron between 324 and 386 nt, and encodes a polypeptide of 770 amino acids with a deduced molecular weight of 83.06 kDa and theoretical isoelectric point (pI) of 5.93. A phylogenetic tree was constructed using CgAS and 25 sequences with maximum similarity obtained from the NCBI database, and phylogenetic analysis demonstrated that CgAS clustered with sequences from *C. tenue*, MPI-CAGE-AT-0009, and *C. fimeti* belonging to the genus *Chaetomium* and phylum *Ascomycota*, shared a similarity of 93.77 %–98.44 % (Fig. 1). Subsequently, multiple sequence alignments and secondary structure element projections were performed to identify the conserved regions and structural characteristics of CgAS and its orthologous proteins (Fig. S1). Secondary structure predictions showed that CgAS contains 27 *α*-helices and 31 *β*-sheets, exhibits high degree of homology with the top 10 closest relationship species, comprises multiple phosphorylation sites, and plays a critical role in numerous physiological and pathological processes in cells. In addition, analysis of the structure of CgAS proteins in various filamentous fungi clearly demonstrated that most of these proteins exhibited a distinctive association with the characteristic PabA (anthranilate/para-aminobenzoate synthase component II) and IGPS (indole-3-glycerol phosphate synthase) domain, which could serve as a catalytic domain with a high degree of homology. These proteins could be structurally distinguished by the presence of a PRAI subunit (phosphoribosylanthranilate isomerase), which can catalyze the conversion of N-(5′-phosphoribosyl)-anthranilate to 1-(*o*-carboxyphenylamino)-1-deoxyribulose 5-phosphate in tryptophan biosynthesis (Fig. 1). The results of in silico analyses of CgAS revealed the presence of multiple functional structural domains with a high level of similarity to those of other filamentous fungi, implying that CgAS possibly has a critical role in tryptophan biosynthesis.

### 3.2 Development of genetic operating system and evaluation of constitutive promoter strength in C. globosum W7

**Fig. 2:**
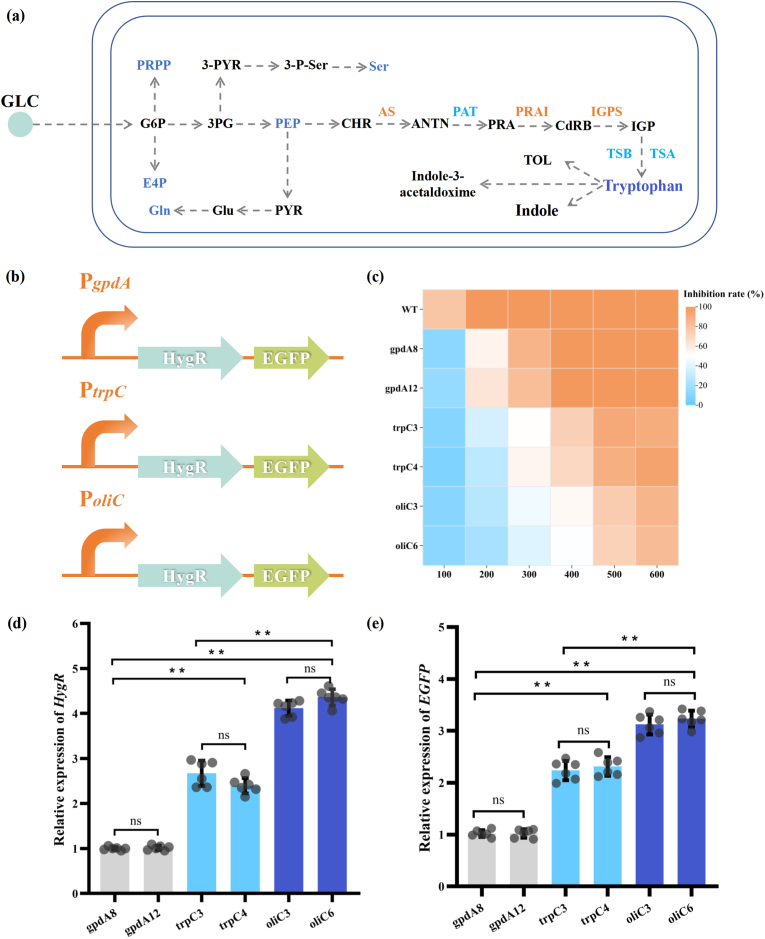
Schematic diagram of tryptophan biosynthetic pathways and heterologous promoter strength verification in *C. globosum* W7. (a) Schematic representation showing steps involved in tryptophan biosynthetic; (b) Plasmid construction strategies used for promoter strength detection in target cells; (c) Heatmaps illustrating drug susceptibility profiles of all detection species. Hygromycin tolerance was measured in PDA medium at 28 °C for 6 days; (d–e) Quantitative estimation of *gpdA* and *EGFP* genes in the mutants, which was manipulated by the heterologous promoter. The transcription levels of target genes in gpdA8 was employed as the standard (which were arbitrarily assigned a value of 1.0) for statistical analysis of the expression levels in detective species. The mean ± standard deviation for six biological replicates are presented. The levels of significance are ∗∗*p* < 0.01, ∗*p* < 0.05. Abbreviations: GLC: Glucose; G6P: glucose-6-phosphate; PRPP: phosphoribosyl pyrophosphate; E4P: erythrose-4-phosphate; 3 PG: glycerate-3-phosphate; PEP: phosphoenolpyruvate; 3-PYR: 3-phosphonooxypruvate; 3-P-Ser: 3-phosphoserine; Ser: serine; PYR: pyruvate; Glu: glutamate; Gln: glutamine; CHR: chorismate; ANTN: anthranilate; PRA: N-(5′-phosphoribosyl)-anthranilate; CdRB: 1-(*o*-carboxyphenylamino)-1-deoxy-ribulose 5-phosphate; IGP: indole-3-glycerol phosphate; TOL: Tryptophol; AS: anthranilate synthase; PAT: phosphoribosyl anthranilate transferase; PRAI: phosphoribosyl anthranilate isomerase; IGPS: indole-3-glycerol phosphate synthase; TSA: tryptophan synthase *α* subunit; TSB: tryptophansynthase *β* subunit.

Bioinformatics analysis indicated that *CgAS* encodes a critical gene for tryptophan biosynthesis, and that an increase in the expression level of the target gene might contribute to the enhancement of tryptophan production and metabolism, thereby providing sufficient precursor for cheA biosynthesis in *C. globosum* W7 (Fig. 2a). To manipulate the expression of the *CgAS* gene, the strength of various heterogeneous promoters was first analyzed in chassis cells. Accordingly, plasmids containing hygromycin resistance and a green fluorescent screening marker, regulated by different constitutive promoters (*gpdA*, *trpC,* and *oliC*), was constructed. The schematic of vector construction is presented in Fig. S2 and 2b, and the details of the promoter sequence are listed in Table S3. The pBlueScript SK was utilized as the skeleton vector, and the building blocks, along with the candidates and selective markers, were connected using seamless splicing technology. To assess the accuracy of the transformants, diagnostic PCR was performed using primer pairs (promoter-TF1/promoter-TR1 and promoter-TF2/promoter-TR2) designed based on the *HygR* gene, spanning the upstream and downstream regions of the insertion site in the pBlueScript SK. The 1366-, 1205-, and 906-bp DNA fragments were accurately amplified from the various derivatives using promoter-TF1/promoter-TR1 (Fig. S2b). When validated with another pair of primers, all the transformants produced a consistent 1034-bp fragment (Fig. S2c). Subsequently, the obtained PCR-amplified fragments were sequenced and the results confirmed 100 % identity with the template. Double enzyme digestion showed that all the mutants of the three detected carriers produced two distinct bands, thereby confirming the accurate linkage of the skeleton vector to the target sequences (Fig. S2d). The resulting plasmids containing different initiating elements, designated as pSK-*oliC*-HygREG, pSK-*gpdA*-HygREG, and pSK-*trpC*-HygREG, were then introduced into the protoplasts of *C. globosum* W7, following experimental procedures previously described [30,31].

The constructed mutants gpdA8, gpdA12, trpC3, trpC4, oliC3, and oliC6 were cultured under the aforementioned conditions. The genetically modified strains olic3 and olic6, regulated by the *oliC* promoter, exhibited the highest hygromycin resistance. At 100 μg/mL hygromycin concentration, the growth of each tested strain was minimally affected, when compared with that of the wild-type strain, which exhibited an 80 % inhibition rate (Fig. 2c). However, when the hygromycin concentration was increased to 300 μg/mL, species gpdA8 and gpdA12 exhibited significant decline in their viability. Furthermore, at high hygromycin concentration (400 μg/mL), strains trp3 and trp4 presented approximately 90 % inhibition rates, whereas strains oliC3 and oliC6 showed 72 % and 75 % inhibition rates, respectively. Subsequently, the strength of the promoters at the transcription level was further quantified using RT-qPCR. The results demonstrated that the transcription levels of the *HygR* gene in the oliC3 and oliC6 mutants were up to 4.12- and 4.36-fold higher than that in strain gpdA8 (arbitrarily assigned a value of 1.0), which were in agreement with the antibiotic tolerance and fluorescence microscopy results. The transcription levels of derivatives mediated by the *trpC* promoter were 2.67- and 2.40-fold higher than that in the control (Fig. 2d), and the variation trend was positively correlated with the expression level of the target gene. In addition, green fluorescence signals (Fig. S3) were detected across all tested strains, indicating that the individual promoters can accurately and efficiently initiate the expression of green fluorescent protein. Furthermore, the expression intensity of different promoters in *C. globosum* W7 was further verified based on the transcription level of the *EGFP* gene. As shown in Fig. 2e, the expression levels of the mutants under the control of the *oliC* promoter were the highest (3.12- and 3.23-fold), followed by those regulated by the *trpC* promoter (2.24- and 2.31-fold). These results clearly demonstrated that the *oliC* promoter presented optimal initiation capability in the chassis cells, and hence was selected as an essential element for target gene expression optimization in subsequent experiments.

### 3.3 Effects of CgAS overexpression on tryptophan production

**Fig. 3:**
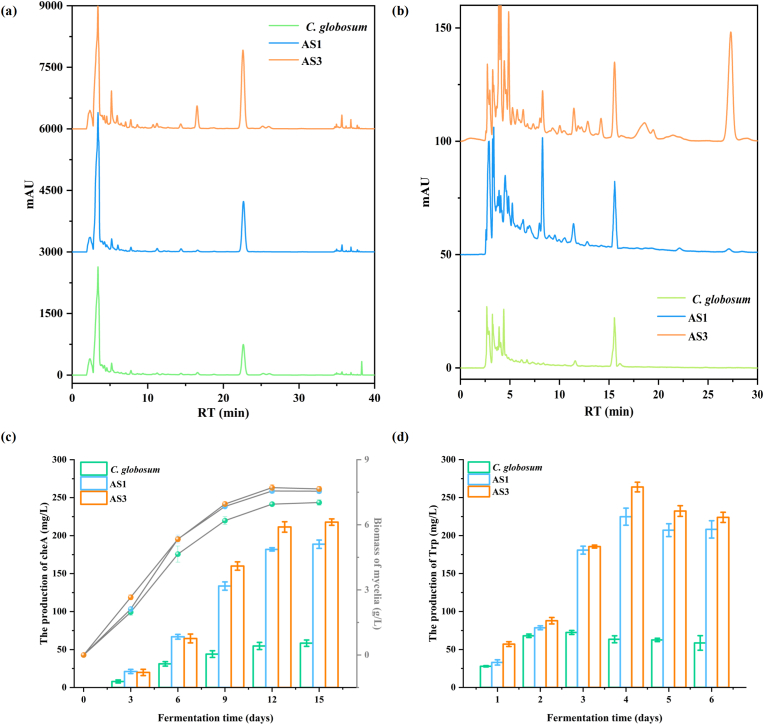
The effects of the *CgAS* gene on tryptophan and cheA production. Detection of cheA (a) and tryptophan (b) production extracts from various derivatives were monitored at 227 and 280 nm by HPLC analysis. (c) Growth curves of tested isolates were obtained by cultivating in PDA medium, and sampling at three days intervals. Biomass is expressed as dry cell weight. Quantitative cheA production of detective strains cultured in fermentation medium. (d) Quantitative tryptophan yield of detective species incubated in PDA fermentation medium. Error bars show standard deviations.

The *CgAS* overexpression vector was constructed using the high-efficient promoter, *oliC* (Fig. S4), and the transformants were verified by diagnostic PCR and sequencing analyses. To investigate the relationship between *CgAS* expression level and target metabolite production in *C. globosum* W7, the genetically transformed strains were cultured in PDA medium over varying fermentation periods and the tryptophan yields were monitored by HPLC. As illustrated in Fig. 3b and d, the yield of tryptophan, a crucial primary metabolite, linearly increased in all the *C. globosum* W7 strains during the initial stage of incubation. This trend was particularly evident in the *CgAS* overexpression mutants AS1 and AS3, which exhibited a remarkably increase in tryptophan production from 64.47 mg/L in the wild-type strain to 224.86 and 263.91 mg/L in the *CgAS* gene-modified transformants after 4 days of incubation, which were 3.54- and 4.16-fold higher than that noted in the control strain, respectively. However, the tryptophan production in all the tested strains gradually declined with time, although the yields remained at higher levels in the genetically modified strains. These observations suggest that the expression of *CgAS* is essential for the biosynthesis of tryptophan and accumulation of precursors.

### 3.4 Role of tryptophan accumulation on cheA production and expression of genes associated with cheA biosynthesis

The use of an efficient promoter, *oliC*, to induce *CgAS* overexpression resulted in significant accumulation of tryptophan during the early stage of fermentation, providing sufficient precursor for subsequent cheA biosynthesis. Investigation of the fermentation profiles of the wild-type strain and genetically modified mutants showed that the cheA concentration in the wild-type strain exhibited an upward trend at the initial three detection time points, and eventually plateaued and stabilized within the range of 54.76–58.43 mg/L following incubation periods exceeding 9 days. In contrast, the production of cheA in AS1 and AS3 was considerably improved throughout the course of 3–12 days of fermentation, with 3.23- and 3.73-fold increase (188.74 and 217.81 mg/L, respectively) in cheA yield, respectively, when compared with that noted in the wild-type strain during the stationary phase (Fig. 3a and c). These findings suggested a strong correlation between the rapid accumulation of tryptophan precursors during the initial stages of fermentation and considerably higher cheA yield in *CgAS*-modified derivatives.

**Fig. 4:**
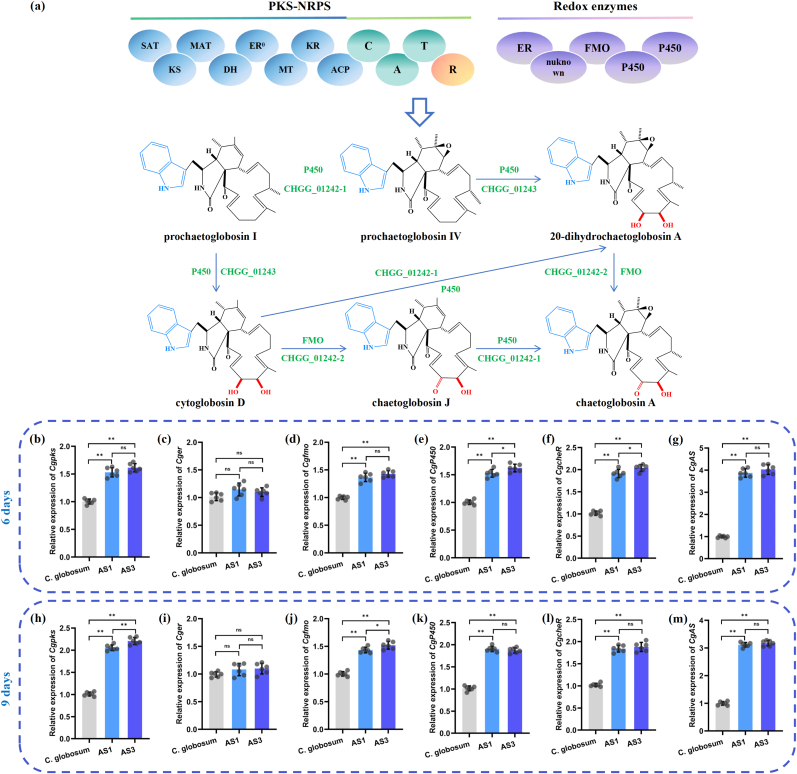
Modular composition of cheA biosynthetic gene cluster and the expression levels of related genes in all treatment groups. (a) Biosynthetic gene cluster and metabolic pathway of cheA; Relative expression levels of *Cgpks* (b and h), *Cger* (c and i), *Cgfmo* (d and j), *CgP450* (e and k), *CgcheR* (f and i), and *CgAS* (g and m) in *C. globosum* W7 (wild type), AS1 and AS3 (with CgAS overexpression cassette) after incubated in a PDA medium for 6 and 9 days. ‘∗’ represented the significance differences between *C. globosum* W7 and *CgAS* mutants. (∗∗p < 0.01, ∗p < 0.05).

To further evaluate the impact of precursor accumulation on the yield of target product, qRT-PCR was performed to assess the genes involved in cheA biosynthesis, including *Cgpks*, *Cger*, *Cgfmo*, *CgP450*, *CgcheR*, and *CgAS*. Following incubation of *C. globosum* W7 and its mutants in PDA medium for 6 and 9 days, RNA samples were extracted from the mycelia. As expected, *oliC* induction successfully increased *CgAS* expression. However, in the later stages of fermentation, the *CgAS* expression level presented a decreasing trend, consistent with the results of extracellular tryptophan analysis. It has been reported that tryptophan can get converted into different active substrates that can enter the secondary metabolite production process during the mid-fermentation stage. When compared with the wild-type strain, no distinct alterations in the transcription of *Cger* were noted in the mutants at the two detection time points (Fig. 4). Following 9 days of fermentation, the expression of FAD-dependent monooxygenase encoding gene, *Cgfmo*, slightly enhanced to 143.50 % and 152.00 % in AS1 and AS3, respectively. Furthermore, the expression levels of *Cgpks* and *CgP450*, which are crucial in the development of the carbon framework and modification of the precursors of cheA, were dramatically improved and presented similar profiles in all the *CgAS*-modified mutants, particularly in AS3, exhibiting 2.21- and 1.87-fold increase, respectively, following incubation period exceeding 9 days. In addition, the transcription levels of *CgcheR*, a positive regulator involved in cheA biosynthesis, were also increased in *CgAS*-overexpression mutants, when compared with that in the control. These findings, combined with the results of tryptophan and cheA production, indicated that regulation of precursor supply is an effective and promising strategy to enhance the expression of secondary metabolite biosynthetic gene clusters, thereby achieving high yields of microbial natural products.

### 3.5 Phenotypic assessment of C. globosum W7 and its mutants

**Fig. 5:**
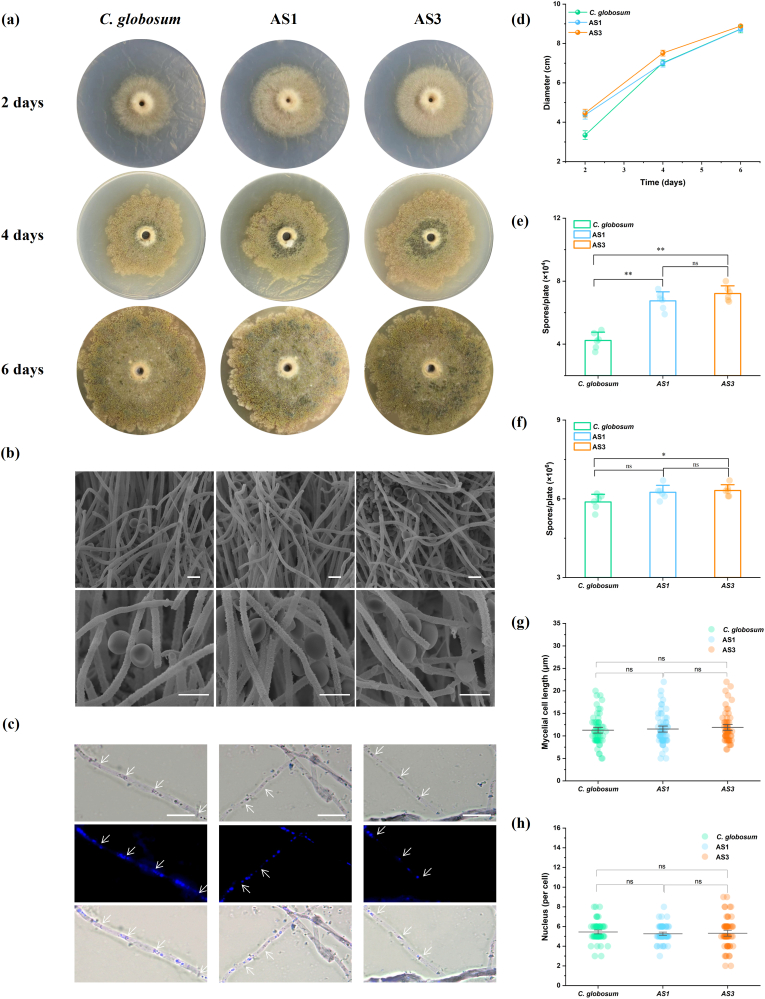
Phenotypic characteristics and morphological differentiation of control strains and *CgAS* mutants. (a) Colony morphology of *C. globosum* W7, AS1, and AS3 incubated on the PDA medium for various times. (b) Morphology characteritics of all detected species after 6 days cultivation observed under the scanning electron microscope. Bar: 10 μm. (c) Observation of mycelial nuclei by DAPI staining. Bar: 10 μm. White arrow indicates mycelial septum; (d) Comparison of fungal development rates of wild-type species and its derivatives; (e) and (f) Spores enumeration of *C. globosum* W7, AS1, and AS3 after 4 and 6 days cultivation observed under the light microscope; (g) Statistics of mycelial cell length; (h) Number of nuclei per cell of wild-type species and *CgAS* overexpession mutants. ‘∗’ represented the significance differences between *C. globosum* W7 and *CgAS* mutants. (∗∗p < 0.01, ∗p < 0.05).

For phenotypic examination, the wild-type and *CgAS*-overexpression strains, which were randomly selected from the obtained transformants, were incubated in PDA medium and then examined under light microscope and scanning electron microscope. As shown in Fig. 5a and b, all the strains showed well-developed aerial and substrate mycelia, with 2.3–3.5-μm diameter, uniform thickness, and distinct verrucose on the surface. Olivaceous-brown, non-motile, lemon shaped (8.3–9.8 × 6.5–7.2 μm) spores with a smooth surface were irregularly arranged on the aerial hyphae, and the number of mycelial nuclei remained unchanged in all the samples, including the control (3–8 nuclei/cell) and genetic derivatives (3–8 and 2–9 nuclei/cell in AS1 and AS3, respectively) (Fig. 5c and h). Similarly, the distribution pattern of mycelial septum spacing in the transformants was consistent with that in the control (Fig. 5g). These findings indicated that the morphological characteristics of *CgAS*-overexpression strains remained unaltered, when compared with those of the parental strain. However, the mutants exhibited improved growth rates, particularly increased spores production in the initial growth phase. Following a 4-days incubation at constant temperature, the wild-type strain produced a spore count of only 4.23 × 10^4^, whereas the *CgAS*-overexpressing transformants exhibited spore counts of 6.75 and 7.22 × 10^4^, respectively. Microscopic observations suggested that the differences in the colony diameter and spores production diminished with the increasing incubation time (Fig. 5d–f). When compared with *C. globosum* W7, the sporulation of AS1 and AS3 increased to 106.23 and 107.37 % after 6 days of incubation, respectively. These findings implied that the *CgAS* gene had no remarkable influences on the morphological characteristics of *C. globosum* W7; however, alterations in its transcription level caused nutrients accumulation, which accelerated fungal cell growth.

### 3.6 Metabolomic analysis of C. globosum W7 and AS3

**Fig. 6:**
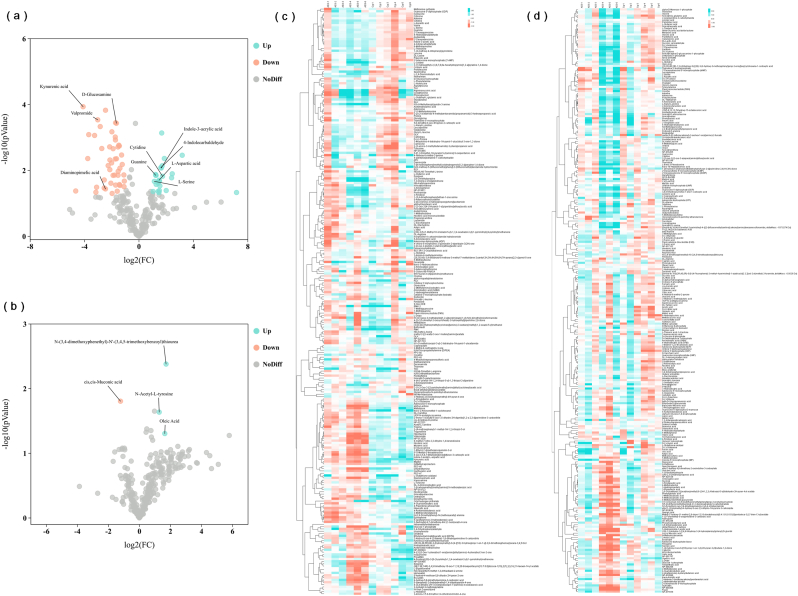
Differentially metabolites analysis of species *C. globosum* W7 and AS3. Differences metabolites volcano plot in positive ion mode (a) or in negative ion mode (b). Each point corresponds to a metabolite, the horizontal-axis (log_2_FoldChange) indicates the multiple changes of each substance in the *CgAS* overexpression mutant compared to the control species, while the longitudinal-axis (-log_10_P-value) represents the statistical significance according to the *t*-test. Metabolites that are significantly down-regulated are marked with orange dots, those that are significantly up-regulated are indicated by blue dots, as well as the gray dots represent metabolites that were detected but not significantly different. Heatmap analysis representing the differentially expressed metabolites in *C. globosum* W7 and its mutant AS3 in positive ion mode (c) and negative ion mode (d).

**Fig. 7:**
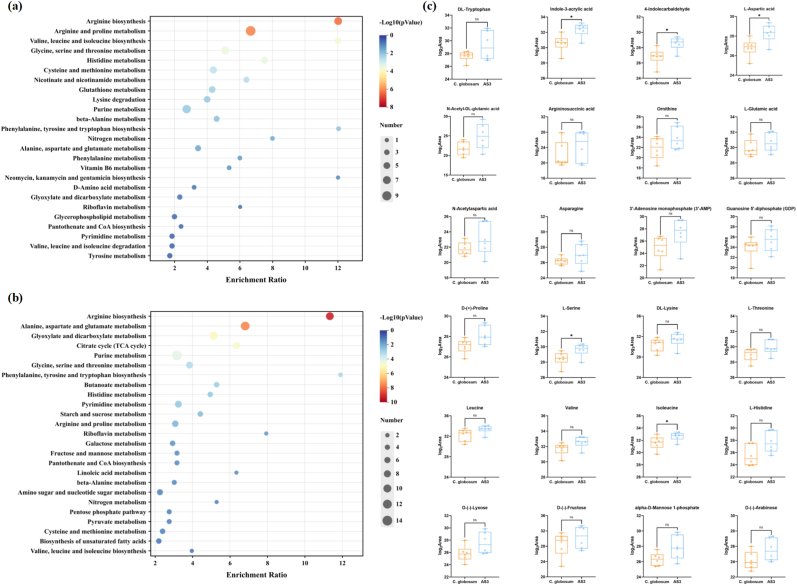
Identification of differential metabolites in strain *C. globosum* W7 after genetic modification. KEGG pathway analysis of differential metabolites (a: positive ion mode; b: negative ion mode). The color of the dots represents the log_10_P-value, and the redder the more significant of the enrichment; (c) Box-plots reveales the differential metabolites in *C. globosum* W7 and AS3. ‘∗’ represented the significance differences between *C. globosum* W7 and *CgAS* mutants. (∗∗p < 0.01, ∗p < 0.05).

To evaluate the metabolic changes in *C. globosum* W7 in response to *CgAS* overexpression, the metabolic profiles of the wild-type *C. globosum* W7 and AS3 were compared through metabolomic analysis in both positive and negative ion modes. The differentially expressed metabolites (DEMs) were identified by LC–MS, and significant differences in metabolite composition were noted between AS3 and wild-type *C. globosum* W7 (Fig. 6c and d, Tables S4 and S5). In the positive ion mode, 85 DEMs were identified, of which 30 DEMs were significantly upregulated and 55 DEMs were significantly downregulated. In contrast, only a few DEMs were detected in the negative ion mode (Fig. 6a and b). KEGG enrichment analysis of DEMs indicated that the changes in the *CgAS* expression level significantly affected “histidine metabolism”, “glutathione metabolism”, “phenylalanine, tyrosine and tryptophan biosynthesis”, etc., with “arginine and proline metabolism”, “purine metabolism”, “arginine biosynthesis”, and “glycine, serine and threonine metabolism” being the most significantly enriched in AS3 and wild-type *C. globosum* W7 (Fig. 7a). Furthermore, the 12 most abundant pathways identified in the negative ion mode included pathways related to “starch and sucrose metabolism”, “pyrimidine metabolism”, “butanoate metabolism”, etc. Among them, pathways associated with “alanine, aspartate and glutamate metabolism”, “Citrate cycle (TCA cycle)”, “arginine biosynthesis”, and “glyoxylate and dicarboxylate metabolism” were the most enriched (Fig. 7b). The enriched DEMs were predominantly associated with amino acid synthesis and metabolism, which could provide sufficient energy source for the development of filamentous fungi and numerous precursors for secondary metabolite production.

For further determination of the variations in microbial metabolism under the influence of *CgAS* overexpression, DEMs were analyzed under the two detection modes. As shown in Fig. 7c, a considerable amount of l-tryptophan was enriched in AS3 via a series of metabolic pathways, which was further converted into indole-3-acetic acid (IAA) and 4-indolecarbaldehyde, the essential intermediates for secondary metabolite biosynthesis. When compared with the wild-type *C. globosum* W7, the IAA and 4-indolecarbaldehyde levels were significantly increased in the *CgAS*-overexpression mutants, with log_2_FC values of 1.64 and 1.61, respectively. In addition, the average levels of ornithine, argininosuccinic acid, N-acetyl-dl-glutamic acid, l-glutamic acid, and l-aspartic acid in the arginine biosynthetic pathway were also increased in the *CgAS*-overexpression mutants, when compared with those in the parental strain. Multiple intermediate products involved in amino acid metabolism displayed similar tendency; in particular, the levels of l-aspartic acid, l-serine, and isoleucine were obviously improved in the *CgAS*-overexpression mutants. Besides, certain numbers of primary metabolites and their related precursors, including d-(−)-fructose, alpha-d-mannose 1-phosphate, 3′-adenosine monophosphate (3′-AMP), etc., which are involved in purine and carbon metabolism, were obviously upregulated in the *CgAS*-overexpression mutants. The changes in l-tryptophan and its derivatives were positively correlated with the accumulation of other amino acids and carbon sources, such as l-aspartic acid, l-serine, isoleucine, d-(−)-fructose, d-(−)-lyxose, d-(−)-arabinose, etc. (Fig. S5). These observations were in accordance with the findings regarding fungal development and cheA production, indicating that the enhancement of nitrogen metabolism increased the supply of energy and precursors in AS3.

## 4 Discussion

It has been reported that cheA, a member of cytochalasin, exhibits strong inhibitory activity against a diverse array of human cancer cell lines, nematodes, and agriculturally important phytopathogenic bacteria and fungi [[36], [37], [38]]. Therefore, research on cheA is particularly significant for plant protection and disease treatment. However, inadequate supply of precursors and low expression levels of crucial genes in cheA biosynthetic gene clusters limit the widespread application of cheA. Although some efforts have been devoted to stimulate the transcription of the cheA biosynthetic gene cluster and generate high-yielding strains by increasing the expression of pathway-specific regulator and certain global regulators or by disrupting the expression of negative regulator [15,16,32,39,40], knowledge about the impact of precursor accumulation on cheA biosynthesis and yield in *C. globosum* W7 remain unclear. In microorganisms, tryptophan is an indispensable amino acid that is required in several physiological processes, such as protein and nucleic acid synthesis, metabolic regulation, signal transduction, and stress resistance [41]. Tryptophan, an aromatic amino acid featuring an indole moiety in the sidechain, serves as a crucial precursor for the biosynthesis of a large number of metabolites with diverse biological activities [42,43]. In particular, cheA, a polyketide alkaloid mainly produced by *C. globosum* W7, requires tryptophan for the backbone assembly. In most of the filamentous fungi, tryptophan is synthesized via five enzymatic steps involving three separate enzymatic components, including AS, phosphoribosylanthranilate transferase, and tryptophan synthase [44,45]. Among them, AS is sensitive to feedback inhibition by tryptophan in the tryptophan biosynthetic pathway [46]. Thus, to enhance tryptophan accumulation, it is essential to overcome the feedback inhibition of AS. The present study aimed to improve cheA yield by enhancing the accumulation levels of its precursor, tryptophan. BLASTP algorithm analysis conducted on the online platform NCBI identified *CgAS* as the putative gene encoding AS in *C. globosum* W7. Phylogenetic analysis showed that CgAS formed a distinct and stable branch with *C. tenue* and clustered with the other two *Chaetomium* spp., MPI-CAGE-AT-0009 and *C. fimeti,* with similarity exceeding 90 %. Multiple sequence alignments and conserved region analysis revealed that CgAS is a putative multifunctional protein containing typical domains (PabA, IGPS, and PRAI) and is highly conversed in filamentous fungi (Fig. S1 and 1). This finding is consistent with previous research on eukaryotic microorganisms, indicating that the tryptophan biosynthetic pathway tends to be less complex with fewer encoding genes in the biosynthetic gene cluster in eukaryotic cell, when compared with that in the prokaryotic cell. A single gene normally consists of multiple functional domains that perform specific functions in the form of multifunctional proteins [44,45].

Characterization of promoter strength is critical for metabolic engineering assay and construction of high-yielding strains. In bacteria and fungi, endogenous and heterologous promoters can be used to accurately regulate the expression of target genes, which is an effective strategy to enhance the production of target metabolites [17,47,48]. However, there is still a lack of understanding regarding the inducible expression capabilities of different heterologous constitutive promoters in *C. globosum* W7. Therefore, in the present study, we first examined the expression strengths of the three constitutive promoters, *gpdA*, *trpC*, and *oliC,* in chassis cells. The results revealed that the heterologous promoters derived from various sources exhibited different degrees of efficacy in *C. globosum* W7. In particular, mutants oliC3 and oliC6, regulated by the *oliC* promoter, displayed the highest level of reporter genes expression, as determined by quantitative analysis (Fig. 2). The established advantages of these heterologous promoters enabled us to strategically modify the cheA biosynthetic pathway in *C. globosum* W7, resulting in an improvement in cheA production and novel products mining. By using the high-efficiency constitutive promoter *oliC*, the AS-encoding gene *CgAS* was constitutively overexpressed in the wild-type *C. globosum* W7. As illustrated in Fig. 3, with the increase in the *CgAS* expression level, the precursor tryptophan content linearly increased at the beginning of fermentation. However, after a 4-days incubation, a declining trend in tryptophan accumulation levels was observed across all the tested strains. This phenomenon can be attributed to multifactorial influences. In the early stages of fermentation, primary metabolism is predominant in the fungus. Subsequently, with the onset of econdary metabolism, substantial quantities of tryptophan, serving as a precursor, are utilized in cheA biosynthesis. Furthermore, tryptophan also serves as a fundamental building block for the synthesis of complex metabolites with diverse structures and biological activities [49,50]. The biosynthesis of all these products in *C*. *globosum* is the predominant reason for the significant decrease in tryptophan production. In addition to its role in secondary metabolite synthesis, tryptophan can be catabolized through two alternative branching pathways: the indole pathway and melatonin pathway. These pathways lead to the formation of various biologically active mediators, including 5-hydroxytryptophan, melatonin, and tryptamine [51,52]. Tryptophan is also catabolized to provide fungal cells with the essential coenzyme nicotinamide adenine dinucleotide (NAD^+^). Indoleamine 2,3-dioxygenase (IDO) is a key enzyme involved in the degradation of tryptophan, a process that is crucial for the synthesis of NAD^+^ via the kynurenine pathway. In *Fusarium graminearum*, three paralogous *FgIDO* genes have been identified, each of which contributes distinctly to the organism's biology processes. Disruption of these genes can lead to varying degrees of NAD^+^ auxotrophy, affecting spore germination, morphological differentiation, and virulence [53]. Therefore, the synthesis and catabolism of tryptophan are crucial for the production of secondary metabolites and proliferation of fungal cells. Moreover, the increase in the relative expressions of *Cgpk*s, *Cgfmo*, *CgP450*, and *CgcheR* further demonstrated that the improvement in the precursor titer could effectively stimulate the production of secondary metabolites. These findings are in agreement with those reported in a previous study on the parasitic fungus *Claviceps purpurea*, in which the tryptophan yield was found to dramatically improve following consistent overexpression of AS-encoding *trpE*, ultimately resulting in a significant increase in ergot alkaloids production [54]. Furthermore, simultaneous overexpression of *OsTDC* together with *OASA1D*, which encode tryptophan decarboxylase and AS, respectively, has been observed to regulate the metabolic flow of tryptophan in rice calli and accumulate potentially pharmacoactive indole alkaloids [55]. These results evidently indicate that AS plays a critical role as the rate-limiting step in tryptophan synthesis, and that the accumulation of precursor tryptophan could efficiently stimulate the production of indole alkaloids.

The production of secondary metabolites in fungi is a complex and diverse process, with the yield of these compounds being influenced by multiple factors. For instance, in the cheA biosynthesis, tryptophan is required as a synthetic precursor. Similarly, the accumulation levels of acetyl-CoA and malonyl-CoA, which serve as carbon chain-extending modular molecules, are also important for the synthesis of the target product [19]. Furthermore, the growth and secondary metabolite synthesis of *C. globosum* are modulated by multiple regulatory factors, forming a complex regulatory network. Various regulators influence cheA production either in a direct or indirect manner. In particular, negative regulators such as CgXpp1 and Cgtf6 have been identified, and the inactivation of their encoding genes can effectively activate the expression of the cheA biosynthetic gene cluster [39,40]. Nevertheless, further investigation is needed to determine whether additional epigenetic regulators or those induced by environmental factors affect cheA biosynthesis. The culture conditions of filamentous fungi can significantly impact the synthesis of their secondary metabolites, and the key environmental factors include ROS, nitrogen and carbon sources, pH, temperature, light, and inter-microbial interactions [[56], [57], [58], [59], [60]]. To enhance the production of cheA, a promising multifaceted strategy can be employed, involving the construction of engineered strains through molecular biology techniques, along with the optimization of culture and fermentation conditions to maximize yield.

Tryptophan is not only involved in the synthesis of multiple bioactive alkaloids, but also provides important metabolic precursors for the biosynthesis of proteins, nucleotides, and cell wall macromolecules. In addition, tryptophan can also significantly affect microbial growth rate, spore production, and morphological differentiation [61,62]. In the present study, phenotypic observations under optical microscope and spore production assays also demonstrated enhanced growth rate, as evidenced by the colony diameter and spore production in AS1 and AS3 (containing *CgAS* overexpression cassette), and the differences between the transformants and the wild-type strain progressively diminished with the increasing incubation time. However, it differed from the inactivation of critical genes involved in tryptophan synthesis, and the morphology of aerial mycelium and spores remained unchanged (Fig. 5) [62]. Furthermore, metabolomic analysis revealed that *CgAS* overexpression significantly affected multiple metabolic pathways, including “arginine and proline metabolism”, “purine metabolism”, “arginine biosynthesis”, “glycine, serine and threonine metabolism”, and “alanine, aspartate and glutamate metabolism” (Fig. 6, Fig. 7), and a considerable amount of l-tryptophan and its derivatives, such as IAA and 4-indolecarbaldehyde, was enriched in AS3 (Fig. 7c). It must be noted that the l-tryptophan derivatives are not only involved in tryptophan metabolism, but also serve as important signaling molecules in microorganisms, contributing to microbial growth, providing protection against adverse conditions, and facilitating the biosynthesis of indole metabolites [63]. Moreover, multiple intermediate products involved in amino acid metabolism presented an upward trend in the mutants, when compared with that in the parental strain; in particular, the levels of l-aspartic acid, l-serine, and isoleucine were obviously increased in the *CgAS*-overexpression mutants. As l-glutamine, phosphoribosyl pyrophosphate, and l-serine are essential precursors for tryptophan synthesis, optimization of l-serine biosynthesis can be advantageous for tryptophan accumulation [64,65]. Besides, l-aspartic acid, as an important intermediate, can be converted into tryptophan and isoleucine through complex enzymatic reactions, and variation in its concentration can affect the synthesis of both these amino acids. In addition, the average levels of certain numbers of primary metabolites associated with purine and carbon metabolism were also clearly enhanced in AS3. In particular, the variations in l-tryptophan and other metabolites, including d-(−)-fructose, d-(−)-lyxose, d-(−)-arabinose, amino acids, and their critical intermediates, were positively correlated (Fig. S5). These results indicated that optimization of tryptophan synthesis contributes to significant enhancement of nitrogen metabolism in microorganisms, which could provide sufficient energy substrates and synthetic precursors for fungal development and secondary metabolite synthesis.

In summary, the present study identified a *CgAS* gene encoding AS involved in the tryptophan biosynthetic pathway, and overexpressed it in *C. globosum* W7 using a promoter optimization strategy. The results obtained indicated that accumulation of precursors could effectively stimulate the biosynthesis of secondary metabolites, providing a novel approach for enhancing the production of biopesticides and clinical drugs.

## 5 Conclusion

Using bioinformatic analysis, this study identified a putative AS-encoding gene, *CgAS,* that catalyzes the rate-limiting step in tryptophan biosynthesis. The *CgAS* gene was then overexpressed under the control of a constitutive promoter *oliC* to improve tryptophan synthesis. As expected, *CgAS* overexpression induced a remarkable increase in tryptophan accumulation in *C. globosum* at the initial stage of fermentation. Besides, *CgAS* overexpression provided sufficient structural units for the generation of the indole alkaloid cheA and improved the cheA-producing capacity of *C. globosum* W7, as evidenced by the cheA titer and transcription levels of the key genes involved in cheA biosynthetic gene cluster. The highly efficient synthesis of tryptophan can drive the generation and transformation of other amino acids, and the active nitrogen metabolism can supply abundant energy for fungal growth and development. Taken together, the present study provides a novel and promising strategy for the accumulation of precursors to optimize antibiotic production.

## CRediT authorship contribution statement

**Shanshan Zhao:** Writing – original draft, Resources, Project administration, Investigation. **Zefei Wang:** Formal analysis, Data curation. **Liyan Tian:** Software, Methodology. **Kejing Li:** Visualization, Validation. **Shiwei Sun:** Methodology. **Gen Chen:** Resources, Methodology. **Daoqiong Zheng:** Writing – review & editing.

## Funding

This study was supported by the Key Research and Development Program of Hainan Province (ZDYF2024SHFZ046), the Fundamental Research Funds for the Central Universities (226-2024-00019), the National 10.13039/501100004731Natural Science Foundation of Zhejiang Province (LDT23D06022D06), the Project of the Donghai Laboratory (Z24ZJ004P), and the Science Foundation of Donghai Laboratory (L24QH014).

## Declaration of competing interest

The authors declare that they have no known competing financial interests or personal relationships that could have appeared to influence the work reported in this paper.
