# Supplementary material for: CgAS, a gene encoding anthranilic acid synthase, contributes to tryptophan biosynthesis and enhanced chaetoglobosin A production in Chaetomium globosum W7
Source: Synth Syst Biotechnol. 2025 May 6;10(3):936–49. doi: 10.1016/j.synbio.2025.05.001 (PMC12139454; doi:10.1016/j.synbio.2025.05.001)
Supplement: Multimedia component 1 [file mmc1.docx]

***CgAS*, a gene encoding anthranilic acid synthase, contributes to tryptophan biosynthesis and enhanced chaetoglobosin A production in *Chaetomium globosum* W7**

**Shanshan Zhao****^a,^****^b,c^, Zefei Wang^b^, Liyan Tian^b^, Kejing Li^b^, Shiwei Sun^b^, Gen Chen^b^, Daoqiong Zheng^b^***

**^a^** Donghai Laboratory, Zhoushan, Zhejiang 316021, China

**^b^** Institute of Marine Biology and Pharmacology, Ocean College, Zhejiang University, Zhoushan, 316021, China

**^c^** School of Life Science and Technology, Harbin Institute of Technology, Harbin 150080, Heilongjiang province, China

* **Correspondence author**:

Email address: zhengdaoqiong@zju.edu.cn

**
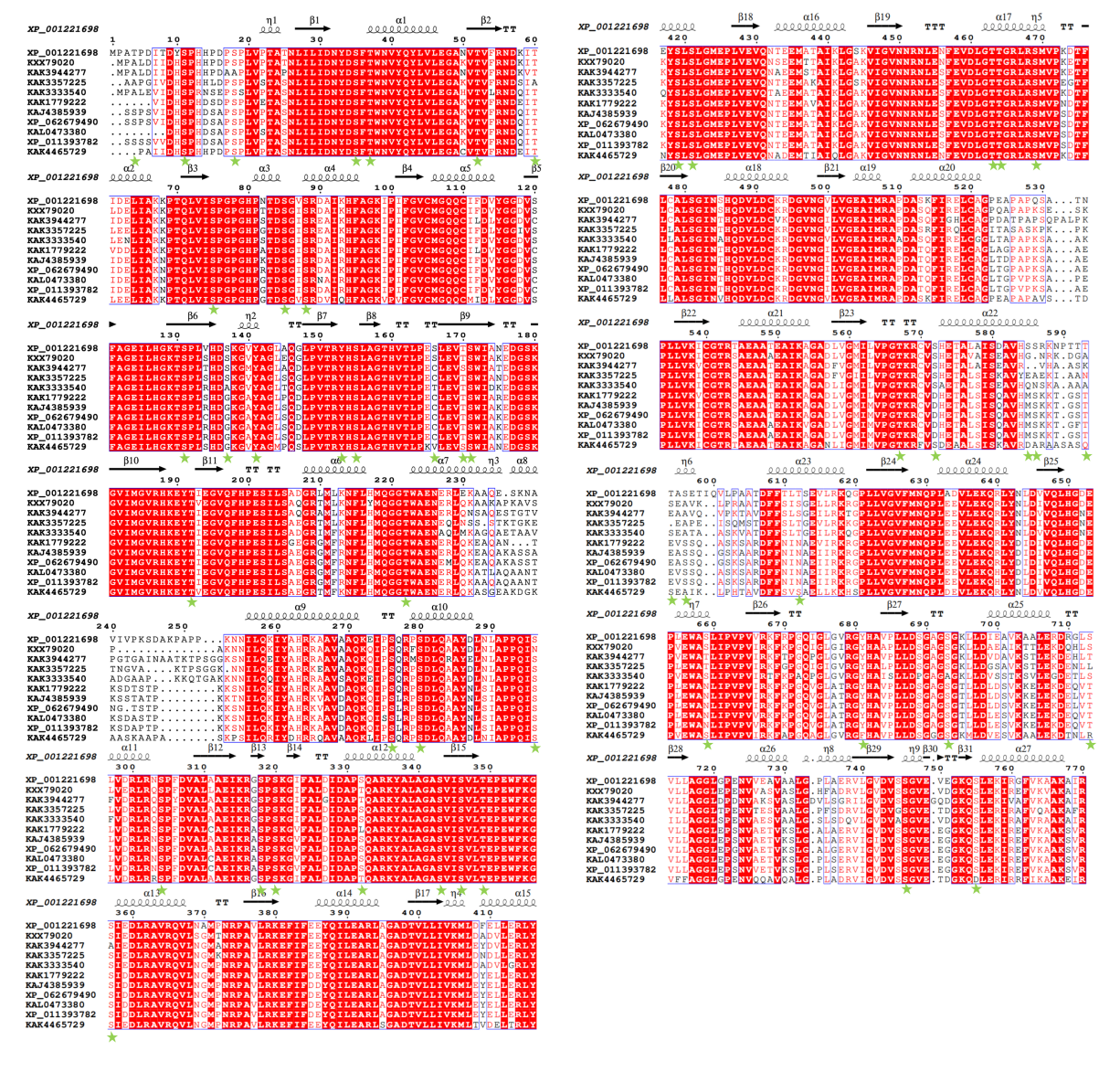
**

**Fig. S1.** Multiple sequence alignment of the CgAS protein sequences. Phosphorylation sites are marked with green pentagram.

**
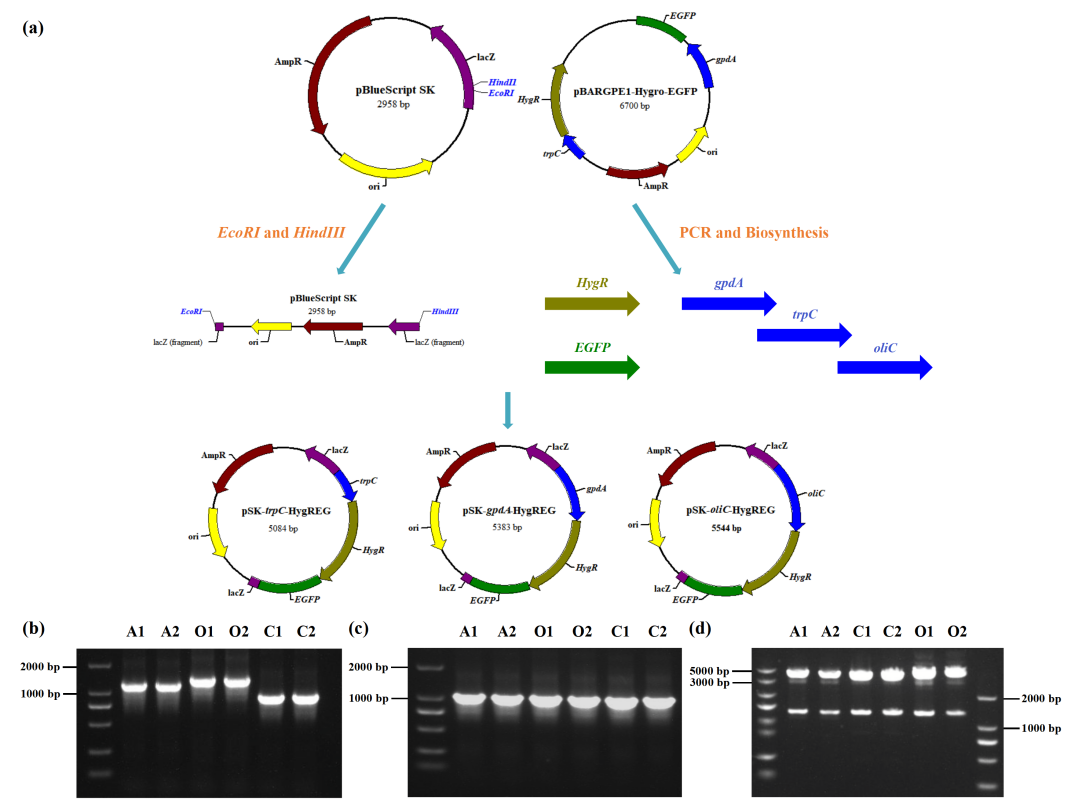
**

**Fig. S2.** Construction and validation of pSK-*oliC*-HygREG, pSK*-gpdA*-HygREG and pSK-*trpC*-HygREG. (a) Construction diagram of carrier pSK-*oliC*-HygREG, pSK*-gpdA*-HygREG and pSK-*trpC*-HygREG; Hygromycin resistance gene (*HygR*) and the fluorescent protein *EGFP* were used as selectable markers. Diagnostic PCR were performed in all mutants by using the pair of primers promoter-TF1/promoter-TR1 (b) and promoter-TF2/promoter-TR2 (c). A1 and A2 were derived from pSK*-gpdA*-HygREG transformation in *DH5α*. O1 and O2 were generated from pSK-*oliC*-HygREG by connecting the linearized carrier pBlueScript SK and markers. C1 and C2 were obtained from pSK-*trpC*-HygREG. (d) Double restriction digestion with *Nde*I and *Eco*RI of all the detected species.

**
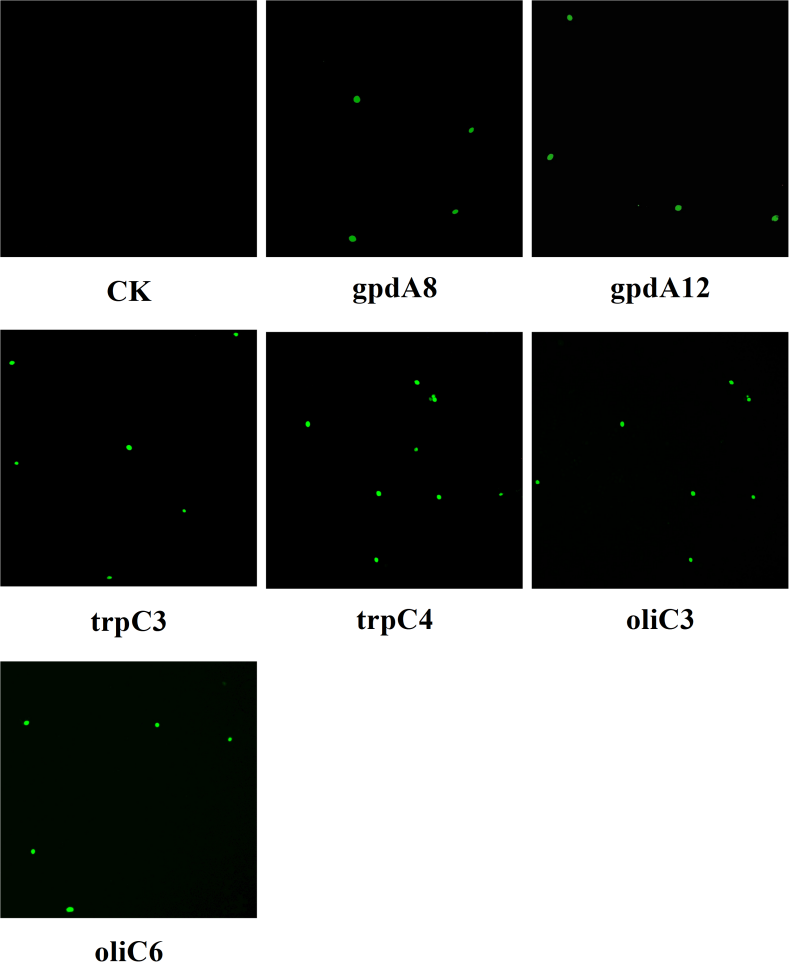
**

**Fig. S3.** Examination of the activated capability of multiple promoters through the expression of EGFP in *C. globosum* W7. CK represented the wild-type species *C. globosum* W7. The mutants gpdA8, gpdA12, trpC3, trpC4, oliC3, and oliC6 contained the promoters for *gpdA*, *trpC*, and *oliC*, respectively.


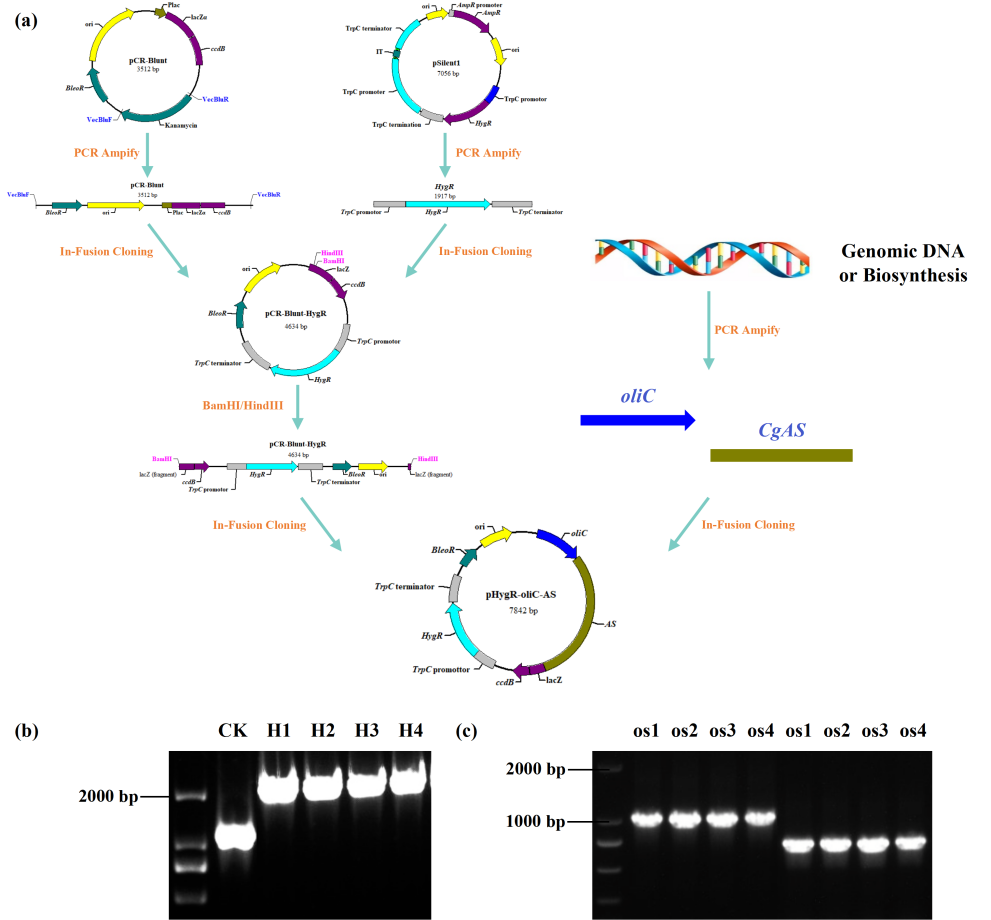


**Fig. S4.** Construction schematic of *CgAS* gene overexpression carrier and transformants verification by diagnostic PCR. (a) Construction diagram of pCR-Blunt-HygR and pHygR-*oliC*-*AS.* Hygromycin (*HygR*) and Bleomycin (*BleoR*) resistance gene were used as selectable marker; Constitutive promoter oliC was employed for expression optimization of target gene; (b) Diagnostic PCR were performed in all mutants by using the pair of primers pKan-TF/pKan-TR. CK was the empty control of vector pCR-Blunt. H1-H6 represented the mutants after plasmid pCR-Blunt-HygR transformation in DH5α; (c) Amplification results of the derivatives from pHygR-*oliC*-*AS* utilizing the pair of primers oliC-AS-F1/oliC-AS-R1 and oliC-AS-F2/oliC-AS-R2. os1-os4 were generated from pHygR-*oliC*-*AS* by connecting the linearized carrier pCR-Blunt-HygR.


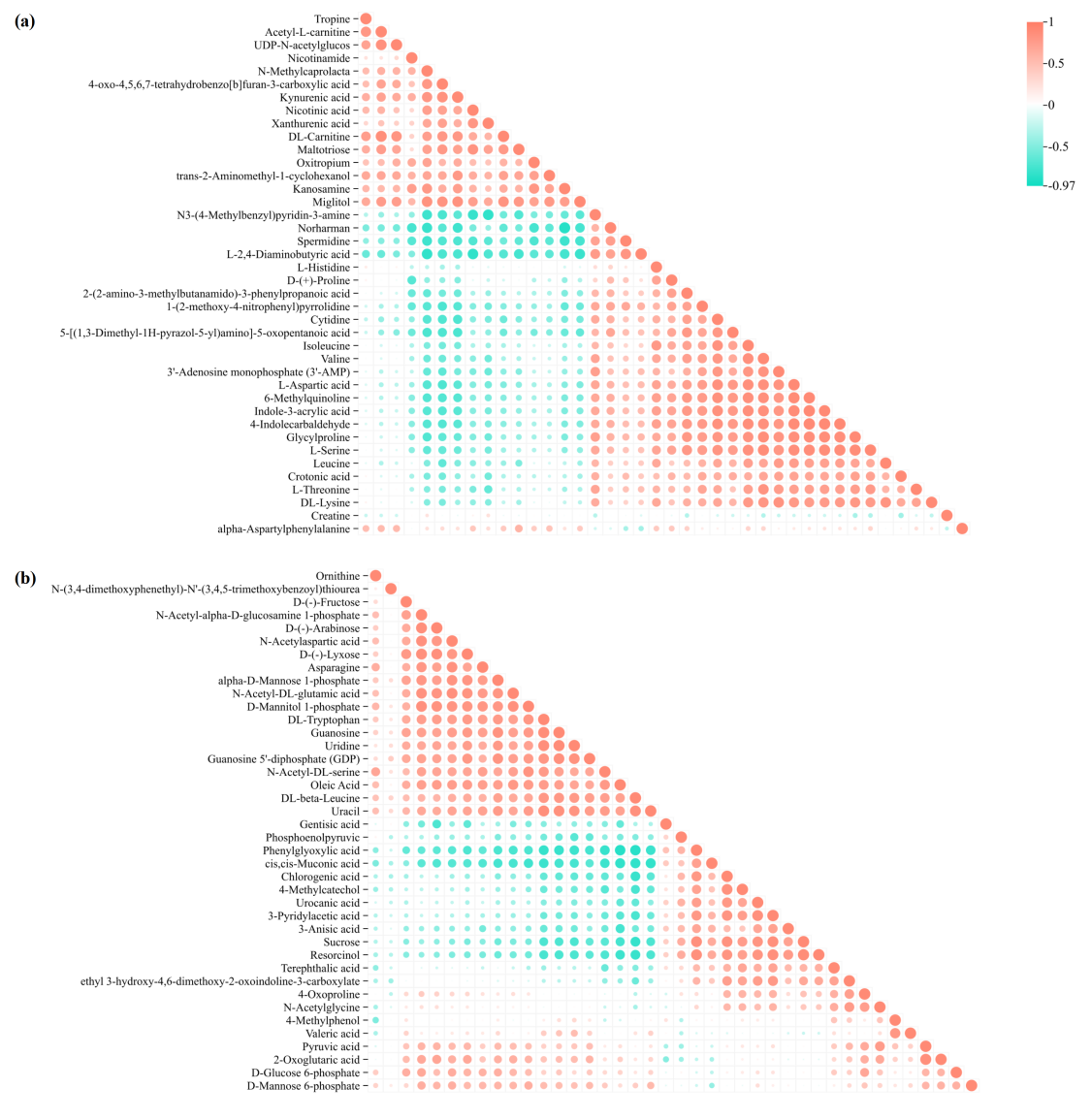


**Fig. S5.** Pairwise correlations of metabolites in in positive ion mode (a) or in negative ion mode (b). Positive correlations are shown in red and negative correlations in green according to Spearman’s rank correlation coefficient.

**Table S1.** Strains and plasmids employed in this study.

| **Strain or plasmid** | **Description** | **Source or reference** |
| --- | --- | --- |
| ***C. globosum* strains** | | |
| *C. globosum* W7 | Parental strain; chaetoglobosin A producer | This study |
| gpdA8 | *C. globosum* derivative (*PgpdA*-*HygR*-*EGFP*) | This study |
| gpdA12 | *C. globosum* derivative (*PgpdA*-*HygR*-*EGFP*) | This study |
| trpC3 | *C. globosum* derivative (*PtrpC*-*HygR*-*EGFP*) | This study |
| trpC4 | *C. globosum* derivative (*PtrpC*-*HygR*-*EGFP*) | This study |
| oliC3 | *C. globosum* derivative (*PoliC*-*HygR*-*EGFP*) | This study |
| oliC6 | *C. globosum* derivative (*PoliC*-*HygR*-*EGFP*) | This study |
| AS1 | *CgAS* gene overexpression mutant | This study |
| AS3 | *CgAS* gene overexpression mutant | This study |
| ***E. coli* strains** | | |
| DH5α | Host strain for cloning | Vazyme Biotech Co., Ltd |
| *Trans*DB3.1 | *ccd*B gene survival competent cell, used to propagate and maintain vectors containing the *ccd*B gene | TransGen Biotech, Beijing, China |
| **Plasmids** | | |
| pBlueScript-SK | Skeleton vector for gene overexpression, contains [ampicillin](javascript:;) (*AmpR*) resistance selectable marker and multiple cloning sites of gene insertion | Miaoling company, Wuhan, China |
| pBARGPE1-*EGFP* | Overexpression vector contains [ampicillin](javascript:;)(*Amp*) and [hygromycin](javascript:;) (*HygR*) resistance gene, the vector involvement of a strong promoter *gpd*A and a green fluorescent protein EGFP selectable marker | Miaoling company, Wuhan, China |
| pSK-*gpdA*-HygREG | Constructed plasmids used for promoter strength verification in *C. globosum*, the vector involvement of a strong promoter *gpdA*, [hygromycin](javascript:;) (*HygR*) resistance gene and a green fluorescent protein EGFP selectable marker | This study |
| pSK-*trpC*-HygREG | Constructed plasmids used for promoter strength verification in *C. globosum*, the vector involvement of a strong promoter *trpC*, [hygromycin](javascript:;) (*HygR*) resistance gene and a green fluorescent protein EGFP selectable marker | This study |
| pSK-*oliC*-HygREG | Constructed plasmids used for promoter strength verification in *C. globosum*, the vector involvement of a strong promoter *oliC*, [hygromycin](javascript:;) (*HygR*) resistance gene and a green fluorescent protein EGFP selectable marker | This study |
| pCR-Blunt | Routine DNA cloning vector contains bleomycin (*BleoR*) and kanamycin (*KanR*) resistance gene, the vector involvement of a *ccdB* lethality gene | Thermo Fisher Scientific, Carlsbad, CA, USA |
| pCR-Blunt-HygR | Skeleton vector for gene manipulate, contains bleomycin (*BleoR*) and [hygromycin](javascript:;) (*HygR*) resistance gene based on pCR-Blunt | This study |
| pHygR-*oliC*-*AS* | *CgAS* gene overexpression vector based on pCR-Blunt-HygR, contains a strong promoter *oliC* and a [hygromycin](javascript:;) resistance selectable marker | This study |

**Table S2.** Primers designed for *C. globosum* W7 in this work.

| **Primer** | **Sequence** |
| --- | --- |
| gpdA-F | 5’- GAGGTCGACGGTATCGATAAGCTTGGGAAAAGAAAGAGAAAAGAAAAGAG -3’ |
| gpdA-R | 5’- CAGGCTTTTTCATGCATGCGGAGAGACGGAC -3’ |
| gpHygRF | 5’- CTCTCCGCATGCATGAAAAAGCCTGAACTCACCGC -3’ |
| gpHygRR | 5’- CCTTGCTCACCATCTATTTCTTTGCCCTCGGACGAG -3’ |
| gpdA-EGF | 5’- GCAAAGAAATAGATGGTGAGCAAGGGCGAG -3’ |
| gpdA-EGR | 5’- GTGGATCCCCCGGGCTGCAGGAATTCTTACTTGTACAGCTCGTCCATG -3’ |
| oliCF | 5’- TCGAGGTCGACGGTATCGATAAGCTTCTGCAGCTGTGGAGCCG -3’ |
| oliCR | 5’- CAGGCTTTTTCATGGATCGATTGTGATGTGATGGA -3’ |
| oliC-HF | 5’- CACAATCGATCCATGAAAAAGCCTGAACTCACCGC -3’ |
| oliC-HR | 5’- CCTTGCTCACCATCTATTTCTTTGCCCTCGGACGAG -3’ |
| oliC-EF | 5’- GCAAAGAAATAGATGGTGAGCAAGGGCGAG -3’ |
| oliC-ER | 5’- GTGGATCCCCCGGGCTGCAGGAATTCTTACTTGTACAGCTCGTCCATGCC -3’ |
| trpC-HygF | 5’- TCGAGGTCGACGGTATCGATAAGCTTTCGACAGAAGATGATATTGAAGGAGC -3’ |
| trpC-HygR | 5’- CCTTGCTCACCATCTATTTCTTTGCCCTCGGACGAG -3’ |
| trpC-EF | 5’- GCAAAGAAATAGATGGTGAGCAAGGGCGAG -3’ |
| trpC-ER | 5’- GTGGATCCCCCGGGCTGCAGGAATTCTTACTTGTACAGCTCGTCCATGCC -3’ |
| promoter-TF1 | 5’- GCCAGTGAATTGTAATACGACTCA -3’ |
| promoter-TR1 | 5’- ATGTAGTGTATTGACCGATTCCTTG -3’ |
| promoter-TF2 | 5’- GGACCGATGGCTGTGTAGAAG -3’ |
| promoter-TR2 | 5’- CGCAACGCAATTAATGTGAGTTAG -3’ |
| pBSK-GF | 5’- GTTTTAAATCAATCTAAAGTATATATGAGTAAACTTGGTCT -3’ |
| pBSK-GR | 5’- AATTACCCCGTAGAAAAGATCAAAGGATCTTCTT -3’ |
| trpC-GF | 5’- TATACTTTAGATTGATTTAAAACTCGACAGAAGATGATATTGAAGGAGCA -3’ |
| trpC-GR | 5’- CTAGGAATTAATTGGTTACTTCCTAATCGAAGCTTTGCTTG -3’ |
| SKG418-F | 5’- TAGGAAGTAACCAATTAATTCCTAGGCCACCATGTTGG -3’ |
| SKG418-R | 5’- GATCCTTTGATCTTTTCTACGGGGTAATTGCGGGACTCTAATCATAAAAACCCA -3’ |
| BSKG-TF1 | 5’- GGTAAGCCCTCCCGTATCGTAGTT -3’ |
| BSKG-TR1 | 5’- CAGTCATAGCCGAATAGCCTCTCCA -3’ |
| BSKG-TF2 | 5’- GCGATGCCTGCTTGCCGAATA -3’ |
| BSKG-TR2 | 5’- GGCGGTGCTACAGAGTTCTTGAA -3’ |
| oliC-AS-F1 | 5’- GAATACTCAAGCTATGCATCAAGCTCTGCAGCTGTGGAGCCG-3’ |
| oliC-AS-R1 | 5’- GGGTGGCAGGCATGGATCGATTGTGATGTGATGGAG-3’ |
| oliC-AS-F2 | 5’- CACAATCGATCCATGCCTGCCACCCCAGA-3’ |
| oliC-AS-R2 | 5’- CACTGGCGGCCGTTACTAGTGGATCCTACCGAATAGCCTTCGCCG-3’ |
| oliC-AS-TF1 | 5’- CGGCTCGTATGTTGTGTGGAATTG -3’ |
| oliC-AS-TR1 | 5’- ACACCGTCACATTGGCACCTTC-3’ |
| oliC-AS-TF2 | 5’- GTACAACCTCGACGTGGTCCAG-3’ |
| oliC-AS-TR2 | 5’- CACAAACAGACGATAACGGCTCTCT-3’ |
| VecBluF | 5’- ATTATTAACGCTTACAATTTCCTGATGCG -3’ |
| VecBluR | 5’- GCGAAACGATCCTCATCCTGTCTC -3’ |
| HygRFor | 5’- AGAGACAGGATGAGGATCGTTTCGCCCACCGCGGTGGCGG -3’ |
| HygRRev | 5’- TCAGGAAATTGTAAGCGTTAATAATAACCCAGGGGCTGGTGAC -3’ |
| pKan-TF | 5’- AAAACGCAAGCGCAAAGAGAAA -3’ |
| pKan-TR | 5’- GAAAAATAAACAAATAGGGGTTCCG -3’ |
| *actin*-F | 5’-TCATCGACAATGGCTCCGGTATG-3’ |
| *actin*-R | 5’-GCTCGTTGTAGAAGGTGTGATGC-3’ |
| RT-*cheR*-F | 5’-CCGCCGCGATACGATGACTT-3’ |
| RT-*cheR*-R | 5’-GCGTTCTGGTGCCGATTTGC-3’ |
| RT-*AS*-F | 5’-GGTGTCAGCCGTGATGCCAT-3’ |
| RT-*AS*-R | 5’-CGTGGACCAGCGGTGATGTT-3’ |
| RT-ER-F | 5’-GTCGGGAGTTTGGGCATTCA-3’ |
| RT-ER-R | 5’-ACACCTCGTCTGCGCCATAA-3’ |
| RT-FMO-F | 5’-ACTTCGCCGCCGAATCATCC-3’ |
| RT-FMO-R | 5’-AGCAGAATGGGCAGGGCAAA-3’ |
| RT-PKS-F | 5’-CCGATTGACGGCAAAGGC-3’ |
| RT-PKS-R | 5’-GCAGCAATGGCGAGAGGAA-3’ |
| RT-P450-F | 5’-CGGGGGTGCTTTTTTACAT-3’ |
| RT-P450-R | 5’-GGGTTGACTCGGGCTGACT-3’ |
| qEGFP-F | 5’-AGTGCTTCAGCCGCTACCC-3’ |
| qEGFP-R | 5’-GATGCCGTTCTTCTGCTTGTC-3’ |
| qHygR-F | 5’-ACGGTGTCGTCCATCACAGTTTGCC-3’ |
| qHygR-R | 5’-TTCCGGAAGTGCTTGACATTGGGGA-3’ |

**Table S3.** The details of promoter sequence.

| **Name** | **Length** | **Sequences** | **G + C content (%)** |
| --- | --- | --- | --- |
| *gpdA* | 685 bp | GGGAAAAGAAAGAGAAAAGAAAAGAGCAGCTGGTGGGGAGAGCAGGAAAATATGGCAACAAATGTTGGACTGACGCAACGACCTTGTCAACCCCGCCGACACACCGGGCGGACAGACGGGGCAAAGCTGCCTACCAGGGACTGAGGGACCTCAGCAGGTCGAGTGCAGAGCACCGGATGGGTCGACTGCCAGCTTGTGTTCCCGGTCTGCGCCGCTGGCCAGCTCCTGAGCGGCCTTTCCGGTTTCATACACCGGGCAAAGCAGGAGAGGCACGATATTTGGACGCCCTACAGATGCCGGATGGGCCAATTAGGGAGCTTACGCGCCGGGTACTCGCTCTACCTACTTCGGAGAAGGTACTATCTCGTGAATCTTTTACCAGATCGGAAGCAATTGGACTTCTGTACCTAGGTTAATGGCATGCTATTTCGCCGACGGCTATACACCCCTGGCTTCACATTCTCCTTCGCTTACTGCCGGTGATTCGATGAAGCTCCATATTCTCCGATGATGCAATAGATTCTTGGTCAACGAGGGGCACACCAGCCTTTCCACTTCGGGGCGGAGGGGCGGCCGGTCCCGGATTAATAATCATCCACTGCACCTCAGAGCCGCCAGAGCTGTCTGGCGCAGTGGCGCTTATTACTCAGCCCTTCTCTCTGCGTCCGTCCGTCTCTCCGCATGC | 56.65 % |
| *trpC* | 386 bp | TCGACAGAAGATGATATTGAAGGAGCACTTTTTGGGCTTGGCTGGAGCTAGTGGAGGTCAACAATGAATGCCTATTTTGGTTTAGTCGTCCAGGCGGTGAGCACAAAATTTGTGTCGTTTGACAAGATGGTTCATTTAGGCAACTGGTCAGATCAGCCCCACTTGTAGCAGTAGCGGCGGCGCTCGAAGTGTGACTCTTATTAGCAGACAGGAACGAGGACATTATTATCATCTGCTGCTTGGTGCACGATAACTTGGTGCGTTTGTCAAGCAAGGTAAGTGAACGACCCGGTCATACCTTCTTAAGTTCGCCCTTCCTCCCTTTATTTCAGATTCAATCTGACTTACCTATTCTACCCAAGCAAAGCTTCGATTAGGAAGTAACC | 46.11 % |
| *oliC* | 846 bp | CTGCAGCTGTGGAGCCGCATTCCCGATTCGGGCCGGATTGGTCAAGATTTGCGTCCGAGGTGCCGTCTATCATTCTAGCTTGCGGTCCTGGGCTTGTGACTGGTCGCGAGCTGCCACTAAGTGGGGCAGTACCATTTTATCGGACCCATCCAGCTATGGGACCCACTCGCAAATTTTTACATCATTTTCTTTTTGCTCAGTAACGGCCACCTTTTGTAAAGCGTAACCAGCAAACAAATTGCAATTGGCCCGTAGCAAGGTAGTCAGGGCTTATCGTGATGGAGGAGAAGGCTATATCAGCCTCAAAAATATGTTGCCAGCTGGCGGAAGCCCGGAAGGTAAGTGGATTCTTCGCCGTGGCTGGAGCAACCGGTGGATTCCAGCGTCTCCGACTTGGACTGAGCAATTCAGCGTCACGGATTCACGATAGACAGCTCAGACCGCTCCACGGCTGGCGGCATTATTGGTTAACCCGGAAACTCAGTCTCCTTGGCCCCCGTCCCGAAGGGACCCGACTTACCAGGCTGGGAAAGCCAGGGATAGAATACACTGTACGGGCTTCGTACGGGAGGTTCGGCGTAGGGTTGTTCCCAAGTTTTACACACCCCCCAAGACAGCTAGCGCACGAAAGACGCGGAGGGTTTTGGTGAAAAAAGGGCGAAAATTAAGCGGGAGACGTATTTAGGTGCTAGGGCCGGTTTCCTCCCCATTTTTCTTCGGTTCCCTTTCTCTCCTGGAAGACTTTCTCTCTCTCTCTTCTTCTCTTCTTCCATCCTCAGTCCATCTTCCTTTCCCATCATCCATCTCCTCACCTCCATCTCAACTCCATCACATCACAATCGATCC | 52.84 % |

**Table S4.** Metabolites identified that were differentially abundant between *C. globosum* W7 and AS3 in the positive ion mode.

| **Name** | **Formula** | **m/z** | **FC** | **p-value** | **log2(FC)** | **log10(p-value)** | **Trend** | **KEGG ID** |
| --- | --- | --- | --- | --- | --- | --- | --- | --- |
| DL-Carnitine | C7 H15 N O3 | 162.11243 | 0.301914866 | 0.00118956 | -1.727786299 | 2.924613589 | down |  |
| Leucine | C6 H13 N O2 | 132.10182 | 1.872070747 | 0.062044078 | 0.904634956 | 1.207299663 | up | C00123 |
| Glycylproline | C7 H12 N2 O3 | 173.09203 | 5.270543155 | 0.016735417 | 2.397951646 | 1.776363452 | up |  |
| L-Histidine | C6 H9 N3 O2 | 156.07671 | 1.383334187 | 0.17905275 | 0.468149726 | 0.747019005 | up | C00135 |
| L-Aspartic acid | C4 H7 N O4 | 134.04474 | 2.782294051 | 0.019843707 | 1.476274902 | 1.702377186 | up | C00049 |
| Acetyl-L-carnitine | C9 H17 N O4 | 204.12293 | 0.287962744 | 0.013893456 | -1.796045925 | 1.857189709 | down |  |
| L-Threonine | C4 H9 N O3 | 120.06543 | 2.009093945 | 0.08307352 | 1.006545026 | 1.08053739 | up | C00188 |
| Isoleucine | C6 H13 N O2 | 132.10185 | 1.789635526 | 0.069850665 | 0.839665801 | 1.155829454 | up |  |
| DL-Lysine | C6 H14 N2 O2 | 147.11277 | 2.023303148 | 0.131073902 | 1.016712493 | 0.882483771 | up | C16440 |
| 5'-S-Methyl-5'-thioadenosine | C11 H15 N5 O3 S | 298.09685 | 0.138456672 | 0.000467121 | -2.852493522 | 3.330570528 | down | C00170 |
| Glucose 1-phosphate | C6 H13 O9 P | 261.03697 | 0.28997498 | 0.277612064 | -1.785999669 | 0.556561665 | down | C11450 |
| Cytidine | C9 H13 N3 O5 | 487.17827 | 3.322710868 | 0.015036701 | 1.73236076 | 1.822847428 | up | C00475 |
| L-Glutathione oxidized | C20 H32 N6 O12 S2 | 613.15912 | 0.033665867 | 0.196308111 | -4.892569556 | 0.707061756 | down | C00127 |
| Indole-3-acrylic acid | C11 H9 N O2 | 188.07053 | 3.111146801 | 0.008445237 | 1.637446471 | 2.073388142 | up |  |
| 3'-Adenosine monophosphate (3'-AMP) | C10 H14 N5 O7 P | 348.07036 | 6.205682779 | 0.05008743 | 2.633589951 | 1.300271254 | up | C01367 |
| UDP-N-acetylglucosamine | C17 H27 N3 O17 P2 | 608.08888 | 0.347060319 | 0.010627605 | -1.526741671 | 1.973564605 | down | C00043 |
| D-Glucose 6-phosphate | C6 H13 O9 P | 261.03696 | 0.980241746 | 0.949212168 | -0.028790506 | 0.022636703 | down | C00092 |
| Ornithine | C5 H12 N2 O2 | 133.0971 | 0.238920867 | 0.024425883 | -2.065395233 | 1.612149724 | down | C01602 |
| L-Serine | C3 H7 N O3 | 106.04975 | 2.187324462 | 0.021445443 | 1.129167242 | 1.668664988 | up | C00065 |
| Cystathionine | C7 H14 N2 O4 S | 223.0747 | 2.217630697 | 0.400023042 | 1.149019133 | 0.397914992 | up | C00542 |
| Adenosine 5'-monophosphate | C10 H14 N5 O7 P | 348.07033 | 0.378635634 | 0.008596893 | -1.401117904 | 2.065658499 | down | C00020 |
| Nicotinic acid | C6 H5 N O2 | 124.0392 | 0.298807958 | 0.000970129 | -1.742709521 | 3.013170492 | down | C00253 |
| Cytidine 5'-diphosphocholine | C14 H26 N4 O11 P2 | 489.11465 | 0.369600341 | 0.02677342 | -1.435962009 | 1.572296156 | down | D00057 |
| Guanosine 5'-diphosphate (GDP) | C10 H15 N5 O11 P2 | 444.03157 | 3.652635755 | 0.168031121 | 1.868937894 | 0.774610274 | up | C00035 |
| Imidazoleacetic acid | C5 H6 N2 O2 | 127.05013 | 0.440427245 | 0.107092146 | -1.183024377 | 0.970242377 | down | C02835 |
| Adenosine diphosphate (ADP) | C10 H15 N5 O10 P2 | 428.03667 | 0.279360489 | 0.097741352 | -1.839800107 | 1.009921658 | down | C00008 |
| 3-Aminophenol | C6 H7 N O | 110.05992 | 3.404151343 | 0.115714297 | 1.767295178 | 0.936612979 | up | C05058 |
| Tropine | C8 H15 N O | 142.12261 | 0.224078389 | 0.001928057 | -2.157924579 | 2.714880162 | down | C00729 |
| Spermidine | C7 H19 N3 | 146.16515 | 9.049169057 | 0.003467125 | 3.177785322 | 2.460030497 | up | C00315 |
| D-(+)-Proline | C5 H9 N O2 | 116.0705 | 2.251786276 | 0.074865412 | 1.171069903 | 1.125718779 | up | C00763 |
| Phloroglucinol | C6 H6 O3 | 127.0389 | 6.745327058 | 0.142326863 | 2.753888396 | 0.846713123 | up | C02183 |
| Nicotinamide | C6 H6 N2 O | 123.0552 | 0.344307546 | 0.014016267 | -1.538230293 | 1.853367629 | down | C00153 |
| Creatine | C4 H9 N3 O2 | 132.07668 | 1.003189953 | 0.995754447 | 0.004594805 | 0.001847745 | up | C00300 |
| 4-Indolecarbaldehyde | C9 H7 N O | 146.06001 | 3.052962017 | 0.008198251 | 1.610209641 | 2.086278788 | up |  |
| 4-Acetamidobutanoic acid | C6 H11 N O3 | 146.08115 | 0.412731683 | 0.003406102 | -1.276723904 | 2.467742366 | down | C02946 |
| Prolinamide | C5 H10 N2 O | 115.08649 | 3.169182746 | 0.140714025 | 1.664110853 | 0.851662615 | up | C19781 |
| L-Glutamyl-L-glutamic acid | C10 H16 N2 O7 | 277.103 | 2.646043495 | 0.261958485 | 1.403836777 | 0.581767531 | up | C05282 |
| 4-Hydroxybenzaldehyde | C7 H6 O2 | 123.04396 | 2.388720101 | 0.014165098 | 1.256237815 | 1.84878042 | up | C00633 |
| Propionylcarnitine | C10 H19 N O4 | 218.13864 | 0.304342265 | 0.083804457 | -1.716233397 | 1.076732884 | down | C03017 |
| alpha-Lactose | C12 H22 O11 | 702.26608 | 0.2485444 | 0.005901544 | -2.008424499 | 2.229034333 | down | C00243 |
| N3-(4-Methylbenzyl)pyridin-3-amine | C13 H14 N2 | 199.12298 | 143.7920341 | 0.046806027 | 7.167839945 | 1.329698219 | up |  |
| 4-oxo-4,5,6,7-tetrahydrobenzo[b]furan-3-carboxylic acid | C9 H8 O4 | 181.04948 | 0.073448186 | 0.004362051 | -3.767129336 | 2.360309269 | down |  |
| alpha-Aspartylphenylalanine | C13 H16 N2 O5 | 281.11319 | 0.709961142 | 0.199073011 | -0.494188031 | 0.700987616 | down |  |
| 2-(2-amino-3-methylbutanamido)-3-phenylpropanoic acid | C14 H20 N2 O3 | 265.15468 | 3.235391268 | 0.063594421 | 1.693940193 | 1.196580986 | up |  |
| L-Phenylalanine | C9 H11 N O2 | 166.08617 | 1.190014133 | 0.804098579 | 0.250978708 | 0.094690705 | up | C00079 |
| N-Acetylputrescine | C6 H14 N2 O | 131.11782 | 0.474044497 | 0.021117749 | -1.076905607 | 1.675352383 | down | C02714 |
| 2'-Deoxyguanosine | C10 H13 N5 O4 | 268.10402 | 2.39671833 | 0.026484688 | 1.261060369 | 1.577005146 | up | C00330 |
| Kynurenic acid | C10 H7 N O3 | 190.0498 | 0.057923914 | 0.000116153 | -4.109697107 | 3.934969615 | down | C01717 |
| Acetanilide | C8 H9 N O | 136.07565 | 2.339908657 | 0.013256196 | 1.226452212 | 1.877581098 | up | C02558 |
| Valpromide | C8 H17 N O | 144.13828 | 0.122110951 | 0.000291453 | -3.033735501 | 3.535431895 | down | D02766 |
| Guvacoline | C7 H11 N O2 | 142.08621 | 0.229646154 | 0.241924662 | -2.122515473 | 0.616319857 | down | C16821 |
| Hypotaurine | C2 H7 N O2 S | 110.02692 | 0.207465476 | 0.007142321 | -2.269056813 | 2.14616064 | down | C00519 |
| 3-Methylcrotonylglycine | C7 H11 N O3 | 158.08113 | 0.267693238 | 0.002194547 | -1.901347398 | 2.658655203 | down |  |
| Xanthurenic acid | C10 H7 N O4 | 206.04569 | 0.378843181 | 0.028600986 | -1.400327315 | 1.543618988 | down | C02470 |
| Maltotriose | C18 H32 O16 | 505.17652 | 0.126230384 | 0.065149448 | -2.985868877 | 1.18608926 | down | C01835 |
| Kanosamine | C6 H13 N O5 | 180.0866 | 0.358813535 | 0.001716388 | -1.478693783 | 2.765384634 | down | C12212 |
| Oxitropium | C19 H25 N O4 | 332.1857 | 0.169907887 | 0.019459074 | -2.557175268 | 1.71087784 | down |  |
| 6-Methylquinoline | C10 H9 N | 144.08073 | 3.259903402 | 0.007232228 | 1.704829215 | 2.140727881 | up |  |
| Glycyl-L-leucine | C8 H16 N2 O3 | 189.12331 | 2.67117482 | 0.043020927 | 1.417474399 | 1.366320232 | up | C02155 |
| Miglitol | C8 H17 N O5 | 208.11794 | 0.077190659 | 0.000820026 | -3.695429921 | 3.086172587 | down | C07708 |
| Crotonic acid | C4 H6 O2 | 87.04399 | 2.900841127 | 0.029451039 | 1.536471284 | 1.53089938 | up | C01771 |
| Diaminopimelic acid | C7 H14 N2 O4 | 191.10259 | 0.180924748 | 0.036386182 | -2.466538332 | 1.439063516 | down |  |
| N-Methylcaprolactam | C7 H13 N O | 128.10693 | 0.251493118 | 0.000672954 | -1.991409172 | 3.172014619 | down |  |
| Indole-3-acetyl-L-aspartic acid | C14 H14 N2 O5 | 291.09754 | 0.110070971 | 0.064414191 | -3.183494052 | 1.191018446 | down |  |
| L-Tyrosine | C9 H11 N O3 | 182.08122 | 0.427069174 | 0.059750584 | -1.227458328 | 1.223657844 | down | C00082 |
| Dihydrothymine | C5 H8 N2 O2 | 129.0658 | 0.169415709 | 0.034187726 | -2.561360441 | 1.466129788 | down | C00906 |
| Esculetin | C9 H6 O4 | 211.06003 | 0.106214947 | 0.223183127 | -3.234941287 | 0.651338643 | down | C09263 |
| trans-2-Aminomethyl-1-cyclohexanol | C7 H15 N O | 130.12257 | 0.039769828 | 0.042059177 | -4.652181862 | 1.376139228 | down |  |
| Buflomedil | C17 H25 N O4 | 308.18562 | 0.466837155 | 0.259056918 | -1.099008708 | 0.586604806 | down | D07176 |
| 2-Imino-1-isobutyl-5-oxo-1,5-dihydro-2H-dipyrido[1,2-a:2,3-d]pyrimidine-3-carbonitrile | C16 H15 N5 O | 294.13364 | 0.103937338 | 0.000868793 | -3.266214082 | 3.06108392 | down |  |
| Norharman | C11 H8 N2 | 169.07598 | 3.185322922 | 0.000731261 | 1.671439637 | 3.13592766 | up | C20157 |
| Epinephrine | C9 H13 N O3 | 184.09677 | 0.115231196 | 0.032343817 | -3.117396749 | 1.490208724 | down | C00788 |
| L-2,4-Diaminobutyric acid | C4 H10 N2 O2 | 119.08142 | 5.445317036 | 0.012655901 | 2.445016048 | 1.897706935 | up | C03283 |
| L-Cystine | C6 H12 N2 O4 S2 | 241.03114 | 16.9592581 | 0.134267345 | 4.084001154 | 0.872029597 | up | C00491 |

**Table S5.** Metabolites identified that were differentially abundant between *C. globosum* W7 and AS3 in the negative ion mode.

| **Name** | **Formula** | **m/z** | **FC** | **p-value** | **log2(FC)** | **log10(p-value)** | **Trend** | **KEGG ID** |
| --- | --- | --- | --- | --- | --- | --- | --- | --- |
| Glycolic acid | C2 H4 O3 | 75.00872 | 2.293551089 | 0.159733606 | 1.197583044 | 0.796603703 | up | C00160 |
| Pyruvic acid | C3 H4 O3 | 87.00871 | 0.351179711 | 0.516599848 | -1.509718599 | 0.286845726 | down | C00022 |
| beta-Alanine | C3 H7 N O2 | 88.04035 | 4.152689873 | 0.113431354 | 2.054046134 | 0.945266885 | up | C00099 |
| Thymidine 5'-monophosphate | C10 H15 N2 O8 P | 321.04934 | 2.904844291 | 0.278473543 | 1.538460832 | 0.55521606 | up | C00364 |
| Sucrose | C12 H22 O11 | 341.10884 | 0.548579274 | 0.249596236 | -0.866227978 | 0.602761969 | down | C00089 |
| 5-Aminovaleric acid | C5 H11 N O2 | 116.0717 | 2.816372061 | 0.168719941 | 1.493837936 | 0.772833586 | up | C00431 |
| 4-Oxoproline | C5 H7 N O3 | 128.03527 | 0.548674642 | 0.461416702 | -0.865977194 | 0.335906689 | down | C01877 |
| 2'-Deoxycytidine 5'-monophosphate (dCMP) | C9 H14 N3 O7 P | 306.04974 | 3.763760351 | 0.160044466 | 1.912174771 | 0.795759337 | up |  |
| L-Phenylalanine | C9 H11 N O2 | 164.07162 | 6.84824425 | 0.095248229 | 2.775734158 | 1.021143089 | up | C00079 |
| DL-Alanine | C3 H7 N O2 | 88.04034 | 3.159640636 | 0.1307888 | 1.659760482 | 0.883429446 | up | C01401 |
| Uridine | C9 H12 N2 O6 | 243.06222 | 3.411683374 | 0.096099274 | 1.770483761 | 1.017279894 | up | C00299 |
| 3-Hydroxybutyric acid | C4 H8 O3 | 103.04004 | 0.493785857 | 0.61271349 | -1.018042578 | 0.212742558 | down |  |
| Guanosine 5'-diphosphate (GDP) | C10 H15 N5 O11 P2 | 442.0172 | 6.202870608 | 0.124969986 | 2.632936031 | 0.903194277 | up | C00035 |
| D-Glucose 6-phosphate | C6 H13 O9 P | 259.02238 | 1.460247113 | 0.424155709 | 0.546212532 | 0.372474684 | up | C00092 |
| D-Mannose 6-phosphate | C6 H13 O9 P | 259.02241 | 1.52425829 | 0.376628997 | 0.608107392 | 0.424086246 | up |  |
| L-Aspartic acid | C4 H7 N O4 | 132.03019 | 2.9 | 0.194266385 | 1.5360529 | 0.711602341 | up | C00049 |
| alpha-D-Mannose 1-phosphate | C6 H13 O9 P | 259.02238 | 4.016602587 | 0.087875562 | 2.005975724 | 1.056131886 | up | C00636 |
| Threonine | C4 H9 N O3 | 118.05096 | 5.567346058 | 0.078114114 | 2.476989762 | 1.107270486 | up | C12317 |
| N-Acetyl-DL-glutamic acid | C7 H11 N O5 | 188.05642 | 25.03182616 | 0.172250414 | 4.64569164 | 0.763839725 | up |  |
| L-Histidine | C6 H9 N3 O2 | 154.06217 | 4.515237708 | 0.100805421 | 2.174801942 | 0.996516112 | up | C00135 |
| DL-Tryptophan | C11 H12 N2 O2 | 203.08255 | 6.560216311 | 0.105329619 | 2.713743386 | 0.977449485 | up | C00806 |
| N-Acetyl-alpha-D-glucosamine 1-phosphate | C8 H16 N O9 P | 300.04899 | 5.860679411 | 0.147718004 | 2.551067922 | 0.830566568 | up | C04501 |
| 2-Oxoglutaric acid | C5 H6 O5 | 145.01421 | 0.154489617 | 0.414832099 | -2.694418214 | 0.382127646 | down | C00026 |
| Chlorogenic acid | C16 H18 O9 | 353.08787 | 0.426632358 | 0.220084414 | -1.228934705 | 0.657410712 | down |  |
| DL-beta-Leucine | C6 H13 N O2 | 130.08728 | 5.886443845 | 0.10371917 | 2.557396327 | 0.984140967 | up | C02486 |
| Resorcinol | C6 H6 O2 | 109.02945 | 0.467415824 | 0.083268521 | -1.097221518 | 1.079519151 | down | C01751 |
| S-Adenosylhomocysteine | C14 H20 N6 O5 S | 383.11414 | 7.038866977 | 0.15569947 | 2.815343222 | 0.807712865 | up | C00021 |
| 2'-Deoxyadenosine 5'-monophosphate (dAMP) | C10 H14 N5 O6 P | 330.06093 | 3.765791658 | 0.227548019 | 1.912953185 | 0.642926942 | up | C00360 |
| Xanthine | C5 H4 N4 O2 | 151.02611 | 7.561427129 | 0.112511741 | 2.918658552 | 0.948802155 | up | C00385 |
| Glycine | C2 H5 N O2 | 74.0247 | 5.624510814 | 0.116686142 | 2.491727625 | 0.932980721 | up | C00037 |
| Asparagine | C4 H8 N2 O3 | 131.04619 | 2.34453093 | 0.14216821 | 1.229299312 | 0.847197505 | up | C00152 |
| 2'-Deoxyguanosine 5'-monophosphate (dGMP) | C10 H14 N5 O7 P | 346.05571 | 4.477687342 | 0.158196738 | 2.162753794 | 0.800802476 | up | C00362 |
| Guanosine | C10 H13 N5 O5 | 282.0844 | 3.540243902 | 0.097998779 | 1.823848757 | 1.008779336 | up | C00387 |
| delta-Gluconic acid delta-lactone | C6 H10 O6 | 177.04043 | 4.06194275 | 0.11324335 | 2.022169906 | 0.945987291 | up | C00198 |
| Ornithine | C5 H12 N2 O2 | 131.08257 | 5.093286632 | 0.173208956 | 2.348596909 | 0.761429655 | up | C01602 |
| Malonic acid | C3 H4 O4 | 103.00364 | 6.125117813 | 0.121066955 | 2.614737594 | 0.91697438 | up | C00383 |
| 4-Methylphenol | C7 H8 O | 107.05021 | 0.309059523 | 0.133269541 | -1.694043375 | 0.875269098 | down | C01468 |
| Valeric acid | C5 H10 O2 | 101.06076 | 0.600577876 | 0.228402976 | -0.735576766 | 0.641298242 | down | C00803 |
| Uracil | C4 H4 N2 O2 | 111.01998 | 6.413008651 | 0.164623769 | 2.681001353 | 0.783507459 | up | C00106 |
| D-Mannitol 1-phosphate | C6 H15 O9 P | 261.0381 | 12.83929585 | 0.139599753 | 3.682494178 | 0.855115349 | up | C00644 |
| 3-Anisic acid | C8 H8 O3 | 151.04 | 0.586031746 | 0.173057942 | -0.770949276 | 0.761808466 | down |  |
| N-Acetylaspartic acid | C6 H9 N O5 | 174.04074 | 4.222004699 | 0.137917697 | 2.077928184 | 0.860380005 | up | C01042 |
| N-Acetyl-DL-serine | C5 H9 N O4 | 146.04583 | 6.798016925 | 0.102932836 | 2.765113954 | 0.987446062 | up |  |
| D-(-)-Lyxose | C5 H10 O5 | 149.04553 | 4.172331544 | 0.118180464 | 2.060853803 | 0.92745431 | up | C00476 |
| Pantothenic acid | C9 H17 N O5 | 218.1034 | 3.804733728 | 0.195833369 | 1.927795491 | 0.708113306 | up | C00864 |
| Pseudouridine | C9 H12 N2 O6 | 243.06226 | 4.005363036 | 0.1211393 | 2.001933011 | 0.91671494 | up | C02067 |
| Gentisic acid | C7 H6 O4 | 153.01929 | 0.331486762 | 0.095394315 | -1.592976839 | 1.020477507 | down | C00628 |
| D-(-)-Fructose | C6 H12 O6 | 179.05606 | 4.114370578 | 0.12566337 | 2.040671742 | 0.900791298 | up | C02336 |
| Leucylproline | C11 H20 N2 O3 | 227.14013 | 3.739736842 | 0.160565603 | 1.902936754 | 0.794347485 | up |  |
| Galacturonic acid | C6 H10 O7 | 193.03528 | 4.307331072 | 0.194973611 | 2.106794217 | 0.710024165 | up | D00643 |
| 4-Methylcatechol | C7 H8 O2 | 123.04513 | 0.609725686 | 0.2686412 | -0.713767772 | 0.570827382 | down | C06730 |
| 10-HDA | C10 H18 O3 | 185.11827 | 2.841272778 | 0.344175925 | 1.506537345 | 0.463219512 | up |  |
| L-Alanyl-L-proline | C8 H14 N2 O3 | 185.09313 | 2.896134414 | 0.131815078 | 1.534128562 | 0.88003491 | up |  |
| 1,2,4-Benzenetricarboxylic acid | C9 H6 O6 | 209.00911 | 3.66689381 | 0.210026157 | 1.874558488 | 0.677726614 | up |  |
| Urocanic acid | C6 H6 N2 O2 | 137.03564 | 0.403746935 | 0.087335977 | -1.308476788 | 1.058806818 | down | C00785 |
| Oleic Acid | C18 H34 O2 | 281.2484 | 3.10565635 | 0.049558192 | 1.6348982 | 1.304884546 | up |  |
| Phosphoenolpyruvic acid | C3 H5 O6 P | 166.97514 | 0.541988875 | 0.219457262 | -0.883664855 | 0.658650043 | down | C00074 |
| L-threo-3-Phenylserine | C9 H11 N O3 | 180.0666 | 23.22371968 | 0.108913186 | 4.537527158 | 0.962919538 | up | C03290 |
| 3-Pyridylacetic acid | C7 H7 N O2 | 136.04037 | 0.572819494 | 0.31101669 | -0.803847505 | 0.507216306 | down |  |
| Phenylglyoxylic acid | C8 H6 O3 | 149.02435 | 0.519937598 | 0.088224584 | -0.943589612 | 1.054410381 | down | C02137 |
| Thymidine | C10 H14 N2 O5 | 241.08301 | 3.769399868 | 0.162832591 | 1.914334848 | 0.788258667 | up | C00214 |
| O-Acetylserine | C5 H9 N O4 | 146.04589 | 8.90962384 | 0.110152108 | 3.155364523 | 0.958007188 | up | C00979 |
| Terephthalic acid | C8 H6 O4 | 165.01927 | 0.453144825 | 0.245484233 | -1.141955886 | 0.609976396 | down | C06337 |
| cis,cis-Muconic acid | C6 H6 O4 | 141.01928 | 0.429537768 | 0.015655605 | -1.219143107 | 1.80533015 | down | C02480 |
| 2'-Deoxyinosine | C10 H12 N4 O4 | 251.07731 | 20.00832408 | 0.097300649 | 4.322528426 | 1.011884264 | up | C05512 |
| O-Desmethyl-cis-tramadol | C15 H23 N O2 | 248.16561 | 33.90200836 | 0.148285484 | 5.083298836 | 0.82890136 | up |  |
| N-Acetylglycine | C4 H7 N O3 | 116.03531 | 0.298341648 | 0.478272597 | -1.744962706 | 0.320324502 | down | D03568 |
| Ascorbic acid | C6 H8 O6 | 175.02475 | 9.525445917 | 0.166312113 | 3.251786631 | 0.779076118 | up | D00018 |
| N-(3,4-dimethoxyphenethyl)-N'-(3,4,5-trimethoxybenzoyl)thiourea | C21 H26 N2 O6 S | 433.14231 | 3.290768194 | 0.004175745 | 1.718424405 | 2.379265984 | up |  |
| D-(-)-Arabinose | C5 H10 O5 | 149.0455 | 2.952591996 | 0.118985108 | 1.561982011 | 0.924507392 | up | C00216 |
| ethyl 3-hydroxy-4,6-dimethoxy-2-oxoindoline-3-carboxylate | C13 H15 N O6 | 280.08273 | 0.068291267 | 0.32492559 | -3.872155099 | 0.488216083 | down |  |
| Endothal | C8 H10 O5 | 185.04551 | 8.936793215 | 0.091635486 | 3.159757243 | 1.037936313 | up |  |
| Glutamylhistidine | C11 H16 N4 O5 | 283.10482 | 4.925415142 | 0.142846905 | 2.300245328 | 0.845129164 | up |  |
